# Toward Autonomous Self‐Healing in Soft Robotics: A Review and Perspective for Future Research

**DOI:** 10.1002/aisy.202400790

**Published:** 2025-02-17

**Authors:** Seyedreza Kashef Tabrizian, Seppe Terryn, Bram Vanderborght

**Affiliations:** ^1^ Brubotics Vrije Universiteit Brussel and imec 1050 Brussels Belgium

**Keywords:** autonomous healings, damage closures, damage sensors, self‐healings, soft robotics

## Abstract

Recent advances in dynamic and reversible polymer networks have led to self‐healing soft robots that can restore their physical and electrical properties after damage. However, in most cases, human intervention remains essential for the healing process. This poses a challenge, especially in working environments with limited human access or where human involvement cand hinder efficiency. To address this gap, in this article, first, the different phases of the healing process in soft robotics are discussed and then the technologies that are or can be integrated into self‐healing soft robots to allow each individual phase to be performed autonomously with minimal human involvement are reviewed. Finally, in this article, the challenges of integrating all phases into self‐healing soft robots are discussed and the perspectives on achieving fully autonomous self‐healing in the future are offered. These phases are classified into five: damage detection, damage cleaning, damage closure, stimulus‐triggered material healing, and recovery assessment. Achieving these attributes requires employing physical intelligence at the material level through the use of stimuli‐responsive materials or utilizing embodied intelligence at the system level by integrating healing‐assistive subsystems or a synergistic combination of both. Consequently, self‐healing soft robots can achieve self‐sufficiency in their healing capabilities, rendering them a sustainable solution for broader applications.

## Graphical abstract

In this article, five different phases (damage detection, cleaning, damage closure, material healing, and healing assessment) are investigated to have an autonomous self‐healing at the system level with the focus on soft robotics. It delves into the importance of each phase, why and where they matter, and reviews relevant technologies that enable robotic systems to perform the healing independently, without external assistance.

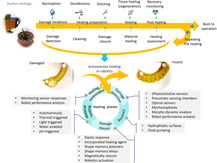

## 1 Introduction

The growing demand for robots designed to dynamically interact and adapt within their environment is increasing in diverse domains, including rehabilitation,^[^ ^1^, ^2^, ^3^ ^]^ prosthesis,^[^ ^1^, ^4^ ^]^ social interactions,^[^ ^5^ ^]^ agriculture,^[^ ^6^ ^]^ object manipulation,^[^ ^7^ ^]^ and inspection tasks.^[^ ^8^, ^9^ ^]^ This necessity highlights the crucial need for the development of soft robots that can ensure safe interactions with both humans and the environment. Soft robots are mainly based on elastic polymers with reversible stretching properties that allow them to absorb and release energy in response to actuation forces, thus facilitating mechanical movements.^[^ ^10^, ^11^ ^]^ However, the inherent softness of these materials renders the robots vulnerable to damage,^[^ ^12^ ^]^ prompting concerns regarding their economic and environmental implications when deployed on a large scale. Fortunately, the emergence of self‐healing materials^[^ ^13^, ^14^, ^15^, ^16^ ^]^ and self‐healing composite materials with enhanced properties,^[^ ^13^, ^17^, ^18^ ^]^ such as improved electrical conductivity, offers promising solutions to address these challenges.

One of the pioneering advancements in the field of self‐healing soft robotics emerged in 2017 with the development of a soft gripper crafted from a Diels–Alder‐based thermoreversible elastomer.^[^ ^19^ ^]^ Subsequent years witnessed a proliferation of self‐healing soft robotic technologies, as extensively cataloged in several comprehensive review articles.^[^ ^12^, ^20^, ^21^, ^22^, ^23^, ^24^ ^]^ These encompass a diverse array of damage‐resilient soft robots founded upon various self‐healing materials and methodologies. Noteworthy approaches include intrinsic self‐healing mechanisms leveraging hydrogen bonding,^[^ ^25^, ^26^, ^27^ ^]^ disulfide bonds,^[^ ^28^ ^]^ Diels–Alder bonds,^[^ ^19^, ^29^, ^30^ ^]^ and supramolecular crystalline phases in biosynthetic materials,^[^ ^31^ ^]^ as well as extrinsic self‐healing strategies using healing agents such as the exploration of UV‐curable thiol‐ene reactions.^[^ ^32^ ^]^ These advancements have demonstrated the capability to mend significant macroscopic damages, including cuts and punctures, thereby restoring the performance of the system.

### 1.1 Healing Phases

**Figure 1:**
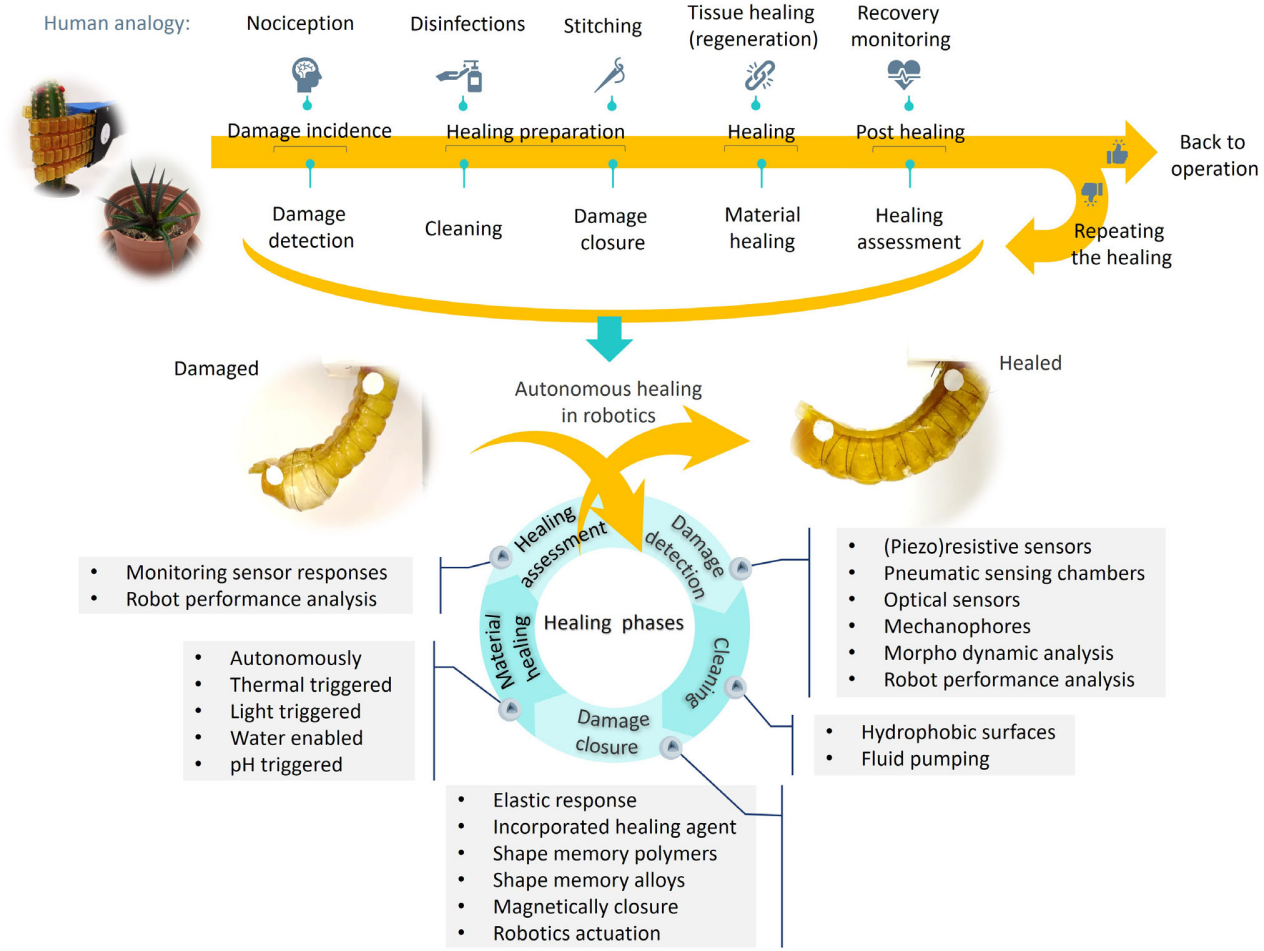
An overview of the autonomous healing phases and the key technologies for each phase is presented. The healing process begins with the detection of damage. Next, the damaged site should be cleaned and closed if there is an open wound. Following this, the material of the robot can undergo the healing process, after which the healing is evaluated. Additionally, there are other strategies for damage resilience, such as adaptation to the damage and compensatory actions after healing, which are discussed at the end of the article.

Nevertheless, successful healing in a soft robotic system involves several phases (**Figure** **1**) and extends beyond the mere self‐healing ability of the material, much like the complex, multi‐phase healing process in the human body^[^ ^21^ ^]^ (Figure 1). i) First, damage must be carefully detected and assessed to plan appropriate subsequent healing actions.^[^ ^33^, ^34^ ^]^ Just as humans perceive pain through the nervous system, robots also require sensory feedback. 2) Cleaning is the next step.^[^ ^34^ ^]^ In case of a severe injuries, we sometimes use antiseptic products for cleaning and disinfection. The body self‐responses as well, by transferring white blood cells to the wound for bacteria destruction and debris removal.^[^ ^35^, ^36^ ^]^ Likewise, in synthetic self‐healing materials, it is crucial to ensure that the damaged site is clean and devoid of dust, dirt, and other impurities that could hinder the efficiency of the healing process.^[^ ^34^ ^]^ 3) The third step involves closing the wound.^[^ ^37^ ^]^ In case of humans, blood clotting and tissue regeneration enable healing of gaping wounds. Moreover, sometimes large wounds are externally closed by, e.g., stitching. Similarly, damage closure in self‐healing soft robots is essential to ensure successful healing, as the synthetic materials cannot grow.^[^ ^37^ ^]^ 4) The fourth step comes to actual healing of the material,^[^ ^19^ ^]^ which is similar to cell regeneration in human body. In synthetic material healing, rebinding of the broken polymer networks or polymerization of a healing agent restore the properties on the material level.^[^ ^12^ ^]^ In some cases, this process occurs autonomously, while in others, an external stimulus such as heat, light, water, or pH changes is necessary to initiate the healing mechanism.^[^ ^12^ ^]^ 5) In the final stage, the health of the system is monitored to assess the effectiveness of the healing process.^[^ ^38^ ^]^ This evaluation serves as a determining factor for deciding whether normal operation can be reinitialized.

### 1.2 Existing Review Papers

Review articles at the self‐healing material level^[^ ^13^, ^14^, ^15^, ^16^, ^17^, ^18^ ^]^ and the self‐healing soft robotics level^[^ ^12^, ^20^, ^21^, ^23^ ^]^ have primarily focused on the fourth phase of healing. Specifically, these reviews examined various self‐healing material chemistries, comparing their mechanical properties, healing efficiencies, and healing times, as well as the application of these materials in robotic systems. Review papers also explored self‐healing stimuli‐responsive polymeric actuators.^[^ ^22^, ^24^ ^]^ However, Tan et al.'s and Terryn et al.'s reviews briefly initiated the discussion on few other phases of the healing process at the system level such as damage detection (pain sensation) and function monitoring.^[^ ^12^, ^21^ ^]^ Liang and Lin shared their perspective on self‐healing control, focusing more on foundational theoretical aspects and taking a broader view beyond just robotics.^[^ ^39^ ^]^ Nonetheless, the comprehensive loop of damage healing in a robotic system that includes all the healing phases discussed in Section 1.1, spanning from the initial detection of damage to the final stage of healing assessment, has not been studied yet.

### 1.3 Self‐Healing Versus Autonomous Self‐Healing

The term 'self‐healing’ refers to a material's ability to recover from damage and restore its compromised properties.^[^ ^13^ ^]^ However, it does not necessarily imply that the self‐healing process occurs autonomously within the system.^[^ ^21^ ^]^ Currently, achieving self‐healing in soft robots typically requires multiple human interventions. The extent of external intervention mainly varies based on the type of self‐healing material used,^[^ ^12^ ^]^ the damage characteristics,^[^ ^37^ ^]^ and the operational condition of the robot.^[^ ^34^ ^]^ The authors believe that this dependence on external interventions is not sufficiently addressed in the current state of the art, which limits the healing process, particularly for envisioned applications beyond human reach, such as flying robots,^[^ ^40^, ^41^, ^42^ ^]^ locomotive robots for exploration,^[^ ^40^, ^43^ ^]^ underwater robots,^[^ ^44^, ^45^, ^46^ ^]^ and human surrogate robots for patient examination during pandemics.^[^ ^47^ ^]^

This article investigates all the pieces of the puzzle, i.e., the healing phases (Figure 1), and delves into the importance of each phase, why and where they matter, and reviews relevant technologies that enable robotic systems to perform these phases independently, without external assistance. Our goal is to accelerate and guide research on autonomous self‐healing in soft robotics, as few existing robots integrate the capabilities needed to execute most healing phases autonomously. Notably, the work of Tang et al. demonstrates a high level of autonomy, incorporating damage sensing and health monitoring, a healing electrofluid capable of sealing large damages, and an integrated heater to polymerize the fluid.^[^ ^48^ ^]^ Other examples exist where robots can independently perform a subset of the healing phases.^[^ ^29^, ^34^, ^37^, ^49^ ^]^ With this work, a better connection between the healing phases and the available technologies can be established, inspiring new research directions. We also discuss challenges of achieving fully autonomous healing in soft robotics and propose a few solutions.

Before discussing each healing phase in detail, we differentiate between autonomous healing at the material level and autonomous healing at the system level. In the domain of self‐healing materials, those that do not need to be triggered for healing are referred to as autonomous self‐healing materials (see Section 5).^[^ ^12^ ^]^ These materials have also been integrated into soft robots^[^ ^50^, ^51^, ^52^, ^53^ ^]^ and cataloged in previous review papers.^[^ ^12^, ^20^, ^54^ ^]^ However, here, autonomous healing refers to the system level, where robots integrate the required technology to perform the healing phases without external intervention. Therefore, the discussion here is not focused on the material's chemistry. Appropriate technologies for each healing phase can be tailored to the robot based on the specific material used. Using autonomous self‐healing materials ensures that the fourth phase of healing is already autonomous (see Section 5).

## 2 Damage Sensing

### 2.1 The Need for Damage Sensing

Soft robots deployed in practical applications, beyond academic settings, are vulnerable to a multitude of damages, including cuts and tears inflicted by sharp objects, bursting due to overloading, as well as issues stemming from fatigue and delamination.^[^ ^12^ ^]^ Hence, following failure, the nature, location, and extent of damage are uncertain. Upon damage, the system should predominantly transition to a healing state,^[^ ^38^ ^]^ ensuring that the damage remains unstressed, and the gap remains closed during the healing process. Consequently, damage detection is inevitable even when working with autonomous self‐healing materials that heal at ambient conditions without an external stimulus.^[^ ^33^, ^34^ ^]^ For soft robots composed of nonautonomous self‐healing materials, which require a stimulus (e.g., light or heat) for healing, damage detection is crucial for initiating or scheduling the stimulus.^[^ ^55^ ^]^

Whereas damage detection offers insights into the general state of the soft robot, information regarding the location and severity of the damage can be essential for the planning of the healing procedure.^[^ ^33^, ^55^, ^56^ ^]^ Depending on these specifics, different actions might be needed to recover the soft robotics performance. By identifying the location of the damage, it may be possible to isolate and unload only the damaged site for healing, using a technology similar to the damage‐adaptive valve by Bosio et al.^[^ ^57^ ^]^ This approach could enable the robot to continue operating during the repair process. Furthermore, soft robots typically have an asymmetric structure, which is the principle behind their actuation and motion in an asymmetric direction.^[^ ^58^ ^]^ This asymmetry originates from the morphology of the robot which can be defined based on different material properties and geometrical design and how these two are arranged together. As such, the loads and the strains are unevenly distributed in the robot structure, leading to some places more vulnerable to damage.^[^ ^33^ ^]^ If the damage happens at a location on the robot that is in tension, immediate healing is necessary to prevent its escalation. Nevertheless, healing of a damage at a location that is not stressed or is in compression can be postponed in case the damage is not fatal to the main function of the robot.^[^ ^33^ ^]^ This strategy can also be generalized to the severity of the damage. Healing of small‐scale damages can be postponed and performed during nonoperational hours of the robot, while detrimental damages need instantaneous healing.^[^ ^37^ ^]^

Moreover, nonautonomous self‐healing materials require healing triggering (see Section 5), which is more efficient when activated exclusively in the damaged area.^[^ ^29^ ^]^ Hence, with knowledge of the damage location, targeted stimulation can expedite the healing process.^[^ ^55^ ^]^ Similarly, damage severity estimation can optimize the healing stimulation process, as the duration and intensity of the stimulation can be modulated based on it. Illustratively, healing of a puncture needs much less healing activation energy in compared with a robot that is cut in half. Additionally, this severity detection affects the cleaning and damage closure actions of the entire healing loop.^[^ ^34^ ^]^ If a large laceration is alarmed, cleaning and damage closure must be checked or the attributed mechanisms should be activated for a successful healing.

### 2.2 Technologies for Damage Sensing

Later, the highlighted damage‐sensing technologies relevant to soft robotics are discussed (**Table** **1**). In addition to these methods, various other sensing techniques have been explored in robotics,^[^ ^59^ ^]^ such as self‐healing capacitive sensors,^[^ ^60^, ^61^ ^]^ piezoelectric sensors,^[^ ^62^ ^]^ and triboelectric sensors.^[^ ^63^ ^]^ However, to the best of the authors’ knowledge, the application of these technologies specifically for damage detection in soft robotics has not yet been a prominent area of study. Furthermore, while damage detection in soft robotics remains in its infancy, it is already a well‐established practice in other fields, such as composite structures used in aerospace, wind turbines, and other industrial applications.^[^ ^64^, ^65^, ^66^ ^]^

**Table 1:** Comparison of different damage detection technologies.

Technology	Principle	Demonstration	Multi‐time detection	Localization	Localization type	Severity	Severity type	Figure	Reference
(Piezo)resistive sensors	Conductive pathway disruption	Flexible electronics	Yes	No	–	No	–	Figure 2b	[72]
	Conductive pathway disruption	Electronic skin	Yes	By integrating different conductive pathways	Discrete	The disrupted pathways count	Discrete	Figure 2a,d	[55, 76]
	Conductive pathway disruption/heat map	Self‐healing heater	Yes	Temperature monitoring by a thermal camera	Continuous	The higher‐temperature area	Continuous	Figure 2c	[29]
	Conductive pathway disruption	Electronic skin	Yes	Distinct overall resistance alteration from each pathway disruption	Discrete	The disrupted pathways count	Discrete	Figure 2g	[33]
	Drift analysis	Electronic skin for actuators	Yes	No	–	The level of drift (not studied)	Continuous	Figure 2h	[75]
	Conductive pathway formation	Electronic skin	No	Those pathways that become conductive	Discrete	The formed pathways count	Discrete	Figure 2e	[56]
	Impedance tomography	Electronic skin	Yes	Impedance distribution	Continuous	The higher impedance area	Continuous	Figure 2f	[67]
Pneumatic chamber	Pressure sudden drop/peak delay analysis	Skin	Yes	Time lag between pressure peaks	Continuous	No	–	Figure 3a	[87]
Optical sensor	Light intensity analysis	Fiber sensor	Yes	No	–	The level of intensity alteration (not studied)	Continuous	Figure 3b	[34]
Mechanophores	Vision (color change)	Actuator	Yes	Monitoring the color change	Continuous	The changed color area	Continuous	Figure 3c	[98]
Morph‐dynamic	Dynamic response analysis	Actuator	Yes	Modeling and response data analysis	Discrete	Modeling and response data analysis (not studied)	Discrete	Figure 3d	[102]
Robot performance analysis	Actuation amplitude analysis	Electronic skin for actuators	Yes	No	–	The level of robot performance alteration (not studied)	Continuous	Figure 3e	[48]
	Vacuum intensity reduction	Universal gripper	Yes	No	–	The level of robot performance alteration (not studied)	Continuous	Figure 3f	[49]

Aside from being sensitive to damage, the damage detection sensors should be selective as well, in particular when using sensors with multiple sensing modalities.^[^ ^33^, ^67^ ^]^ For example, a tactile piezoresistive force sensor responds to both contact forces that cause damage and those that do not, making it challenging to distinguish between a change in resistance resulting from damage and one resulting from touch or force.^[^ ^33^ ^]^ In both cases, a sharp increase in resistance can be observed due to the load, yet it relaxes thereafter, at least when the damage is small. In small damages, the elastic response of the material realigns the indentation caused by the damage agent, recovering the resistance.^[^ ^67^ ^]^ This issue is more pronounced in large‐area sensors.^[^ ^67^ ^]^

#### 2.2.1 (Piezo)resistive Sensors

**Figure 2:**
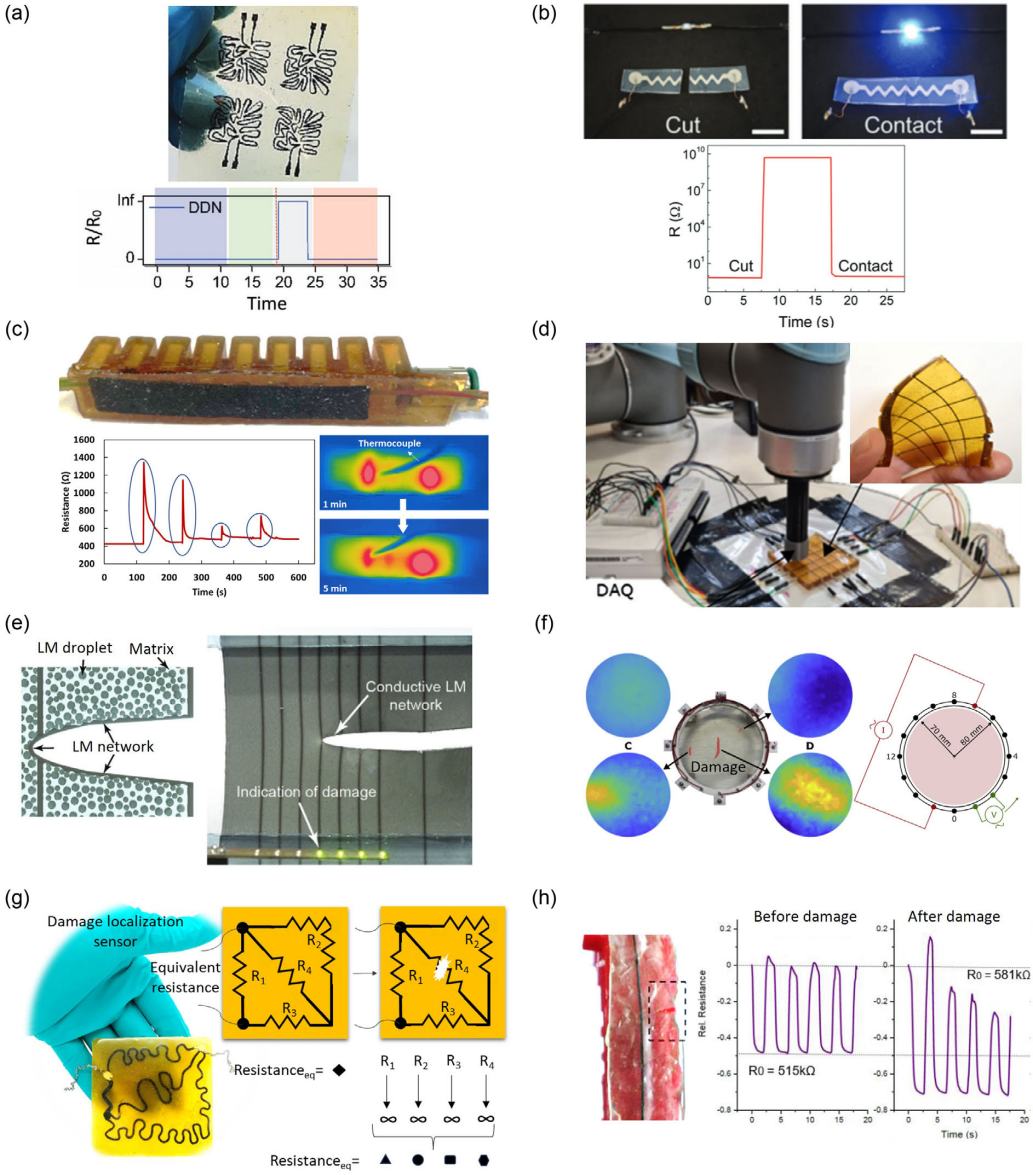
(Piezo)resistive soft damage sensors. a) Self‐healing damage detection sensor skin. Reproduced (Adapted) with permission.^[^ ^55^ ^]^ Copyright 2020, WILEY‐VCH. b) Self‐healing liquid metal–based sensor that can detect damage. Reproduced (Adapted) with permission.^[^ ^72^ ^]^ Copyright 2019, WILEY‐VCH. c) Self‐healing heater that can detect damage with both monitoring the resistance changes and also heat distribution. Reproduced (Adapted) with permission.^[^ ^29^ ^]^ Copyright 2022, IEEE. d) Self‐healing damage localization sensor skin. Reproduced with permission.^[^ ^76^ ^]^ Copyright 2022, WILEY‐VCH. e) Damage localization sensor skin based on liquid metals. Reproduced (Adapted) with permission.^[^ ^56^ ^]^ Copyright 2019, WILEY‐VCH. f) Self‐healing EIT sensor skin. Reproduced with permission.^[^ ^67^ ^]^ Copyright 2023, The Authors, Published by Elsevier Ltd. g) Self‐healing damage localization sensor skin. Reproduced (Adapted) with permission.^[^ ^33^ ^]^ Copyright 2025, IEEE. h) Self‐healing damage sensor skin. Reproduced (Adapted) with permission.^[^ ^75^ ^]^ Copyright 2021, The Authors, Published by MDPI.

The (piezo)resistive effect serves as a prominent sensing principle utilized to enhance perceptual capabilities in soft robots,^[^ ^68^, ^69^ ^]^ as well as in the domain of damage detection.^[^ ^70^ ^]^ When employing a (piezo)resistive sensor for damage detection, it typically assumes a sacrificial role.^[^ ^33^ ^]^ In this context, damage is identified only when the sensor itself is harmed, thereby disrupting the electrical current path and resulting in a significant change in resistance that can be recorded^[^ ^55^ ^]^ (**Figure** **2**a). The restoration of the sensor's damage‐sensing capabilities after healing is crucial for detecting multiple instances of damage. Sensors capable of both detecting damage and completely recovering from it have been developed. In majority of cases, these sensors are fabricated from electrically conductive self‐healing polymers, which can be achieved through either ionic conductivity, commonly found in self‐healing hydrogels,^[^ ^27^ ^]^ or electronic conductivity, typically achieved using conductive fillers in self‐healing elastomeric composites.^[^ ^29^, ^33^, ^55^, ^71^ ^]^ Such filler loaded self‐healing composites usually need to be stimulated for the healing (see Section 5). The addition of conductive fillers, such as carbon‐based nanoparticles or nanotubes, to a soft matrix results in stiffening of the composite material.^[^ ^17^ ^]^ Consequently, piezoresistive sensors based on composites are generally stiffer than the elastomeric material in which they are incorporated, such as the body parts of soft robotics. In many cases, these disparities can lead to delamination between the sensor and the robot due to weak interfacial bonding. However, this issue is mitigated when incorporating self‐healing sensors into a self‐healing soft robot where both the self‐healing composite used for the sensor and the self‐healing polymer used in the soft robot share the same reversible chemistry.^[^ ^61^ ^]^

An alternative approach to achieving self‐healing damage sensors involves utilizing conductive liquids embedded in channels within a self‐healing polymer. This includes self‐healing sensors based on liquid metals^[^ ^56^, ^72^ ^]^ (Figure 2b), or ionic liquids.^[^ ^73^ ^]^ Liquids offer the advantage of inherent self‐healing properties, as any disrupted conductive path can flow back together. This self‐healing process occurs at room temperature and is instantaneous. Consequently, the healing capacity of liquid‐based self‐healing sensors is dependent on the healing capacity of the polymer in which they are embedded.^[^ ^56^, ^72^ ^]^ However, in case of damage, leaking and/or leaching can often pose challenges and require careful consideration.^[^ ^73^ ^]^ Magnetic electrically conductive microparticles can also aid in the recovery of electrical conductivity after damage. This is attributed to the external magnetic field, which helps facilitate better physical contact of the conductive traces, thereby enhancing the restoration process.^[^ ^74^ ^]^

Damage‐detecting (piezo)resistive sensors are ideally integrated into areas of the robot more susceptible to damage, particularly regions where it interacts with the external environment^[^ ^29^ ^]^ (Figure 2c). However, in many applications, predicting the location of damages remains uncertain, necessitating the coverage of large areas of soft robots with damage sensors.^[^ ^75^ ^]^ For example, multiple self‐healing (piezo)resistive sensors can be embedded within a self‐healing polymer to create artificial electronic skins for soft robots^[^ ^76^ ^]^ (Figure 2 d). Khatip et al. have developed a soft self‐healing multilayer electronic skin incorporating a damage detection layer.^[^ ^55^ ^]^ This layer comprises four neuron‐like piezoresistive sensors, constructed from a carbon black–based self‐healing composite. The sensors divide the layer into four regions for discrete damage localization, wherein damage disrupts the conductive pathway of one of the sensors (Figure 2a). Strategic placement of (piezo)resistive sensors across multiple layers, arranged in a grid structure, enhances the localization^[^ ^76^ ^]^ (Figure 2d), i.e., when damage disrupts two or more sensors. Alternatively, Markvicka et al. introduced a skin for damage detection and localization by dispersing liquid metal droplets into a silicon elastomer^[^ ^56^ ^]^ (Figure 2e). They integrated 1D and 2D conductive traces, initially forming an open circuit. Upon skin damage, the rupture of liquid metal droplets establishes a conductive path between the traces, facilitating damage localization.^[^ ^56^ ^]^ Additionally, the number of adjacent traces forming a closed circuit enables the estimation of damage severity. To ascertain the connection between traces, a demultiplexing and multiplexing technique was employed.^[^ ^56^ ^]^ The incorporation of a multilayer approach in electronic skins, wherein multiple damage detection layers are stacked with an insulation layer in between, not only enhances precise damage localization but also enables estimation of damage depth.^[^ ^77^ ^]^

The aforementioned electronic skins exhibit two major limitations: 1) damage localization is restricted to a discrete manner and 2) small damages that do not harm the conductive channels or are not in proximity to a conductive channel cannot be detected. To address these issues, self‐healing electrical impedance tomography (EIT) skins are a promising solution.^[^ ^67^ ^]^ EIT skins are made of (piezo)resistive materials with multiple boundary electrodes.^[^ ^67^ ^]^ A small alternating current is sequentially applied to electrode pairs, while voltages are measured across others. Model or learning‐based algorithms then reconstruct a 2D image of the electrical impedance distribution.^[^ ^78^ ^]^ Changes in impedance due to damage enable continuous detection of location and severity.^[^ ^79^ ^]^ In recent developments, Hardman et al. incorporated ionic conductive self‐healing hydrogels into an EIT skin and characterized it using a model‐free data‐driven approach, enabling the detection, localization, and healing of damages^[^ ^67^ ^]^ (Figure 2f). Similarly, Costa et al. combined an ionic conductive self‐healing hydrogel with a conductive Diels–Alder composite to achieve a self‐healing EIT sensor, allowing for the tuning of local sensitivity.^[^ ^80^ ^]^

In EIT sensors, there are challenges in developing physical or analytical models capable of extracting damage information from vast datasets. Emerging machine learning approaches demonstrate the ability to handle substantial data volumes.^[^ ^81^ ^]^ However, these machine learning techniques require extensive sampling and training of the electronic skin,^[^ ^67^, ^78^ ^]^ potentially incurring large costs. Another challenge with electronic skins featuring numerous electrodes or connectors^[^ ^55^, ^56^, ^67^ ^]^ is their integration into soft robotic systems. The presence of multiple electrodes complicates wiring and assembly, making it a significant barrier for real‐world applications. In a recent work, Kashef et al. have developed an electronic skin capable of localizing damage at four distinct zones using only one pair of electrodes.^[^ ^33^ ^]^ This was achieved by designing the resistance values of each zone to achieve different values of the equivalent resistance of the skin as a result of damage to each zone (Figure 2g).

In contrast, Georgopoulou et al. have explored a dynamic approach for detecting damage in piezoresistive sensing skin for soft actuators,^[^ ^75^ ^]^ wherein damage can be identified even if the conductive part remains undamaged (Figure 2h). This sensor is the result of embedding a self‐healing carbon black–based conductive fiber in a nonconductive self‐healing elastomer with a hydrogen bond structure. Affixed to a tendon‐driven soft actuator, the sensor underwent dynamic testing during bending. The study revealed that a change in the geometrical stiffness of the skin induced by damage leads to a noticeable drift in the signal of the fiber sensor.^[^ ^75^ ^]^ However, dynamic evaluation of the system's health may intensify the extent of the damage. Furthermore, it remains unclear how substantial the damage must be to cause a significant drift in the sensor signal, given that carbon black–based fiber sensors typically experience drift during operation.^[^ ^61^ ^]^ While the method of Georgopoulou et al. cannot pinpoint the location of the damage,^[^ ^75^ ^]^ the severity might be estimated based on the degree of drift in the sensor signal.

Lastly, damage severity and localization can also be monitored using an external device, such as an infrared camera, in combination with (piezo)resistive materials (Figure 2c).^[^ ^29^ ^]^ When (piezo)resistive‐based materials encounter damage that obstructs the normal flow of electrical current, local resistance increases. Application of electrical voltage to the material results in localized heating due to the Joule effect, concentrating heat at the damaged site.^[^ ^82^ ^]^ To the best of the authors’ knowledge, no automated damage localization work has been reported using the combination of infrared cameras (image processing) and (piezo)resistive materials. However, the principle of localized heating due to damage for damage localization can be further exploited by other methods, such as integrating a few temperature sensors in the robot and mapping the temperature distribution with the damage location in soft robots.^[^ ^83^ ^]^

#### 2.2.2 Pneumatic Sensing Chambers

By integrating (micro) channels or air cavities into the flexible structures of robots and connecting them to fluidic pressure sensors, the system gains sensing capabilities. Changes in the volume of these channels or cavities lead to corresponding shifts in pressure. These changes can result from external forces, such as touches (exteroception),^[^ ^30^, ^84^ ^]^ or the robot's movements (leading to proprioception), including bending, stretching, or twisting.^[^ ^85^, ^86^ ^]^ One advantage of these technologies is their unified material structure, allowing the sensor and the robot body to be made from a single material in a single manufacturing step.^[^ ^30^ ^]^ This sets it apart from those sensing methods, like piezoresistive sensors, where mechanical mismatch between the sensor and the main soft body, as well as complex and multistage manufacturing techniques, presents challenges.

**Figure 3:**
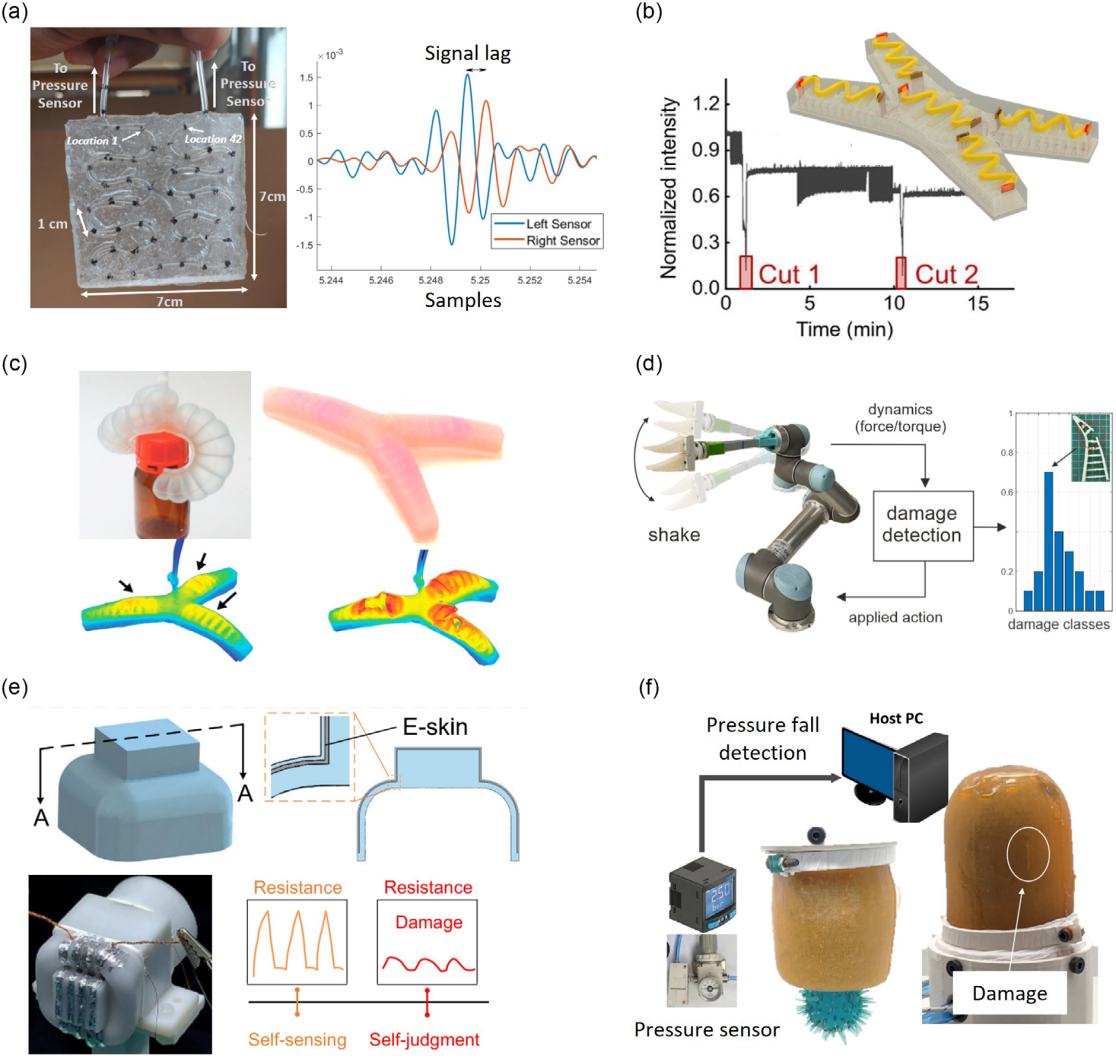
Other damage sensing technologies. a) Pneumatic‐based damage detection and localization self‐healing sensor. Reproduced (Adapted) with permission.^[^ ^87^ ^]^ Copyright 2021, The Authors, Published by MDPI. b) Optical‐based self‐healing damage detection sensor. Reproduced (Adapted) with permission.^[^ ^34^ ^]^ Copyright 2022, The Authors, Published by AAAS. c) Damage observation using mechanophores by Gossweiler et al. Reproduced  (Adapted) with permission.^[^ ^98^ ^]^ Copyright 2015. American Chemical Society. d) Damage detection and localization technique based on morpho‐dynamic analysis. Reproduced with permission.^[^ ^102^ ^]^ Copyright 2022, The Authors, Published by frontiers. e,f) Damage analysis by monitoring the robot's performance. (e) Reproduced (Adapted) with permission.^[^ ^48^ ^]^ Copyright 2023, The Authors, Published by Springer Nature and (f) Reproduced (Adapted) with permission.^[^ ^49^ ^]^ Copyright 2023, The Authors, Published by Wiley‐VCH GmbH.

In addition, pneumatic sensing holds strong potential for damage detection in soft robotics, as illustrated by Thuruthel et al., who developed a pressure‐based damage detection and localization system^[^ ^87^ ^]^ (**Figure** **3**a). They integrated 1D and 2D microchannels into a self‐healing elastomer based on reversible hydrogen bonding, connecting both ends of the channels to separate fluidic pressure sensors. Localization is achieved by measuring the time lag between the pressure profile that is generated by a pressure leak cause by damage.^[^ ^87^ ^]^ Depending on the location of the damage, the time lag shifts, allowing for damage localization. In their 2D sensor, damage is located with a positional accuracy of 2.9 cm,^[^ ^87^ ^]^ which amounts to a localization error within 15% of the length of the sensory pathway. In addition, they demonstrated the self‐healing capacity of the sensor, by fully restoring the sensor performance after multiple damage–detection–healing cycles. Since the restoration of sensing performance depends solely on re‐establishing a hermetic seal, these damage detection sensors heal quickly and completely,^[^ ^87^ ^]^ even if only part of the mechanical properties of their self‐healing polymers are recovered. Alternatively, Kashef Tabrizian et al. demonstrated that the location of external contacts can be estimated by considering variations in the size of the sensor's cavity, resulting in different pressure amplitudes based on the contact location.^[^ ^30^ ^]^ This approach can potentially be used for damage localization as well. However, it is sensitive to the size of the contacting object and also the intensity of the contact.^[^ ^30^ ^]^

In pneumatic sensing chambers, the pressure drop signal due to damage is characterized by its high frequency.^[^ ^87^ ^]^ Consequently, there would be a challenge in differentiation between damage and high‐frequency contacts that do not cause damage. Furthermore, this method solely permits the measurement of the initiation of touch and damage. Once touched or damaged, the location cannot be retrieved anymore.^[^ ^87^ ^]^ Lastly, it can only detect damages that lead to leaking of the channels or cavities. Therefore, superficial cuts or scratches will remain undetected.^[^ ^87^ ^]^

#### 2.2.3 Optical Sensor

Optical fibers serve as versatile sensors capable of measuring various physical quantities such as strain, temperature, pressure, and more. These sensors exploit the modulation of light properties within the fiber (e.g., waveguide), including intensity, phase, polarization, wavelength, or transit time, to gauge the target quantity.^[^ ^88^, ^89^, ^90^ ^]^ They offer a powerful tool for damage detection in a wide range of material and structures, e.g., deeply studied for aerospace composite structures.^[^ ^91^ ^]^ The main components of a fiber optic sensor are the emitter and receiver of the light and the optical waveguides.^[^ ^88^ ^]^ The application defines the required mechanical properties of the waveguides, i.e., in many soft robotics applications, flexibility and stretchability are required. For repeated damage detection, the waveguides must also possess self‐healing capabilities.^[^ ^34^ ^]^ Similar to the pressure‐based sensing, the physics of optics provides inherent heal‐ability. This means that healing of the fiber optic sensor is limited to restoration of the mechanical properties of the self‐healing material used to make the waveguides.^[^ ^34^ ^]^ This stands in contrast to (piezo)resistive sensors, where both the mechanical and electrical properties of the embedded sensing material must be restored.^[^ ^61^ ^]^ The research of Bai et al. is one of the few research articles on this topic, where a soft quadruped is equipped with wavy shape fiber optic damage detection sensors to autonomously adapt its gait based on a damage^[^ ^34^ ^]^ (Figure 3b). The damage is detected based on the sudden drop in light intensity, caused by the external damage agent (e.g., a sharp object) that blocks the light transmission in the waveguide.^[^ ^34^ ^]^ This principle holds promise for expansion in identifying the severity of damage, as varying degrees of damage will modulate the attenuation of light intensity relative to their severity. Moreover, the ability to localize damage can potentially be incorporated into existing optical sensors, inspired by the work of Bai et al. on bending location detection^[^ ^88^ ^]^ and Galloway et al. on collision location detection.^[^ ^89^ ^]^ It should be noted that, similar to pressure‐based sensing, distinguishing between damage and external touch can would present challenges, especially when the fibers are fully pressed but not damaged. Therefore, further investigation is required to attain selective multi‐sensing capabilities.

Apart from optical fibers, the electroluminescent property—i.e., emitting light when subjected to an electric field—of certain materials can also be utilized for detecting damage. Tan et al. developed a self‐healing light‐emitting capacitor and embedded it inside a self‐sealing soft gripper.^[^ ^92^ ^]^ They demonstrated that the luminance and light‐emitting properties of their device were restored after damage. They integrated a light sensor into the gripper to measure the luminance.^[^ ^92^ ^]^

#### 2.2.4 Mechanophores

Inspired by bruising in biological systems, mechanophores are being used to enable materials to visually respond to mechanical stimuli.^[^ ^93^, ^94^, ^95^ ^]^ Mechanophores are molecules that undergo a change in their chemical structure, such as conformational shifts or bond scission,^[^ ^96^ ^]^ when exposed to mechanical stress or strain. These molecules can act as stress/strain or damage indicators because they respond to mechanical forces by producing visible or detectable changes in color or fluorescence.^[^ ^95^, ^96^, ^97^ ^]^

These materials have also been utilized in the creation of gripping and walking soft robots, enabling them to visualize areas subjected to higher strain (potential damage sites)^[^ ^98^ ^]^ (Figure 3c). In another study, limited to the material level,^[^ ^99^ ^]^ a UV fluorescent dye was added to a healing agent stored within hollow microfibers in a composite material. Upon damage, the fibers rupture, releasing the fluorescent healing agent, thereby rendering the damage location visible.^[^ ^99^ ^]^ While the use of mechanophores for damage detection in (soft) robotics has not yet been introduced, the presence of integrated cameras, typically used for motion capture and control in robotic systems, presents opportunities for autonomous damage analysis using image processing with this new class of materials.

#### 2.2.5 Morpho‐Dynamic Damage Sensing

Another damage detection technology is to analyze the response of the structure to a high‐frequency vibration, commonly used to assess infrastructures, such as bridges, buildings, and wind turbines, but also fluidic pipelines for gas or water transport.^[^ ^100^, ^101^ ^]^ If there is a defect in the structure, the system's response deviates from the healthy condition. Similarly, Abdulali et al. conducted a classification study on the soft FinRay gripper to detect and localize changes in the morphology of the FinRay caused by external cuts^[^ ^102^ ^]^ (Figure 3d). The benefit of this technology stems from its sensorless nature, as it does not necessitate any embedded sensor within the soft structure.^[^ ^102^ ^]^ Consequently, this technique does not interfere with the design or fabrication of the soft robotic system, unlike other sensing approaches mentioned earlier, which often require alterations in design or manufacturing methods to embed the sensing element, thereby potentially compromising the robot's performance. However, as stated in the article, the sensing system does not function in real‐time during the operation of the soft robot.^[^ ^102^ ^]^ The robot needs to halt its operation and enter the damage detection phase, which includes shaking the gripper and measuring the force and torque feedback.^[^ ^102^ ^]^ Nevertheless, there would be potential to expand the approach toward morpho‐dynamic damage sensing during operation. For instance, this could involve applying a modulated position trajectory during the travel phase in pick and place applications with soft grippers.

#### 2.2.6 Robot Performance Analysis

Sensors are commonly integrated in (soft) robots for gathering information about both their internal state (proprioceptive sensing) and their surrounding environment (exteroceptive sensing).^[^ ^68^, ^69^ ^]^ Although sensors are typically designed for specific functions other than damage detection, they can serve as sources for identifying damage as well.^[^ ^48^ ^]^ For example, proprioceptive sensors such as stretchable (piezo)resistive sensors can record the robot's positional and/or deformation state in response to input energy.^[^ ^61^ ^]^ Consequently, these sensors can also detect damage if it significantly alters the robot's performance^[^ ^48^ ^]^ (Figure 3e). Wang et al. developed a vacuum‐driven self‐healing universal gripper featuring an integrated pressure sensor to regulate the vacuum level.^[^ ^49^ ^]^ During actuation, potential damage applied to the gripper's membrane induces a change in vacuum pressure inside the membrane, facilitating the detection of damage occurrence^[^ ^49^ ^]^ (Figure 3f). Another approach involves monitoring the robot's performance using red, green, blue, depth and comparing the real‐time performance of the robots with the expected behavior.^[^ ^103^ ^]^ Discrepancies observed between real‐time performance and expected behavior, modeled via finite‐element method modeling, can serve as indicators of potential damage.^[^ ^103^ ^]^

In all scenarios, the detection of damage occurrence is limited to the sensing of significant damages that affect the system's performance.^[^ ^48^, ^49^, ^103^ ^]^ Put simply, it is hardly unlikely to detect damages in a soft robot that do not currently impact its performance but may escalate into more serious issues jeopardizing the robot's functionality. In addition, the authors believe that these approaches strongly rely on the stability of the soft robot toward changes in its environment and the selectivity of the sensor. Without sufficient stability and selectivity, distinguishing performance changes caused by damage from the changes due to external factors like temperature shifts or humidity variation becomes difficult. However, for fluid‐driven soft robots equipped with pressure sensors,^[^ ^49^, ^103^ ^]^ where even minor perforations can significantly reduce system performance through leaks or render the robot nonfunctional, these damage detection approaches are particularly valuable. Further research is warranted to ascertain the efficacy of these methods in providing details about the location and severity of the damage.

## 3 Cleaning

### 3.1 The Need for Cleaning

The presence of dust, impurities, and other contaminants on the surfaces of the exposed damaged area reduces the likelihood of successful healing.^[^ ^34^ ^]^ This primarily occurs because these undesired contaminants create a barrier that impedes the efficient reconnection and subsequent rebinding of the material in the damaged area. Their occurrence varies greatly depending on the specific application in soft robotics, dictating whether a clean‐then‐heal procedure is required. Contamination can be minimized by promptly sealing the damage, a process that can be accelerated through the utilization of damage closure mechanisms outlined in Section 4. Incorporating a cleaning phase can bolster the effectiveness of healing and may serve as a true enabler for certain applications wherein self‐healing soft robots are required to function in contaminated and dusty environments or come into direct contact with dirty or oily objects, potentially causing contamination on their fracture surfaces. Due to the current underestimation of this challenge, further research is required on the cleaning phase to facilitate its advancement and integration into self‐healing soft robots. What might also be interesting is to develop dirt or contamination sensors.

### 3.2 Technologies for Cleaning

**Figure 4:**
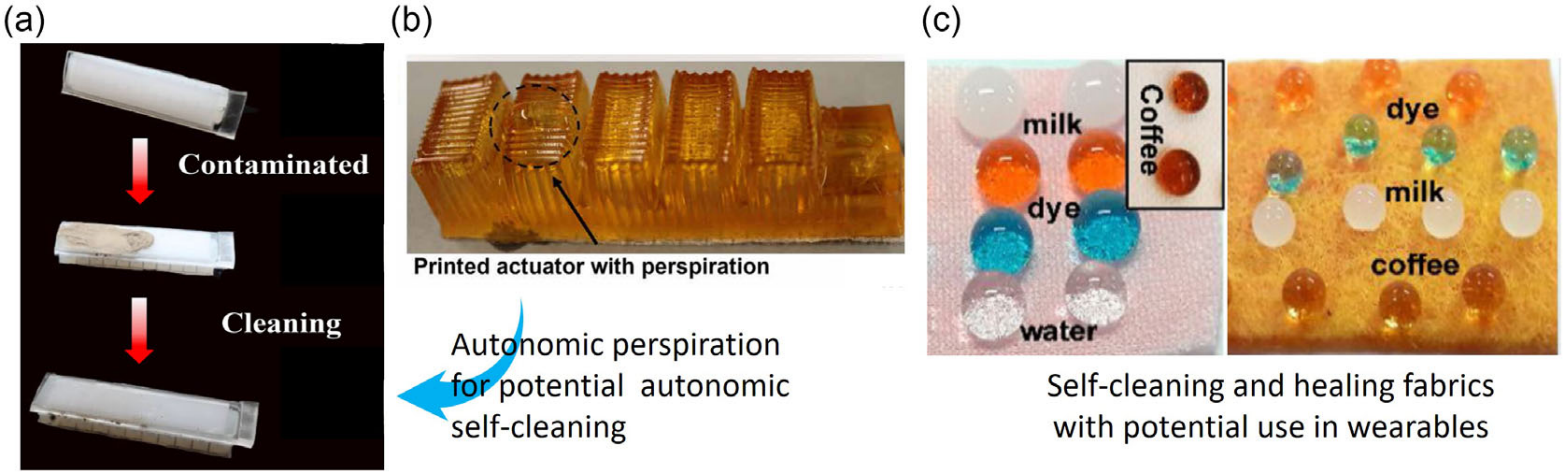
Cleaning‐related works: a) self‐cleaning surfaces with superhydrophobic properties that can remove contamination by water droplets. Reproduced with permission.^[^ ^109^ ^]^ Copyright 2021, Elsevier Ltd. b) Autonomic perspiration in the hydrogel‐based actuator can potentially provide water droplets for cleaning as well. Reproduced with permission.^[^ ^110^ ^]^ Copyright 2020, AAAC. c) Superhydrophobic self‐cleaning and healing fabrics. Reproduced with permission.^[^ ^111^ ^]^ Copyright 2019. Elsevier Ltd. Such fabrics can potentially be used in soft wearable robotic devices.

Inspired by nature,^[^ ^104^ ^]^ self‐cleaning surfaces/coating are under development, mostly onto rigid body objects, e.g., self‐cleaning glasses or aluminum plates.^[^ ^105^ ^]^ This involves engineering the surface chemistry and architecture to achieve a low surface energy and thereby exhibit (super)hydrophobic properties, akin to those found in lotus leaves, rose petals, and butterfly wings.^[^ ^106^, ^107^, ^108^ ^]^ Consequently, the surface repels contaminants by causing them to roll off, often with the assistance of water droplets. Following this principle, Wu et al. developed a self‐cleaning flexible surface and applied it to a soft gripper^[^ ^109^ ^]^ (**Figure** **4**a). They demonstrated that the surface can not only easily clean fine sand pollutants using a small amount of water but also can improve the robot's performance by tuning its friction upon externally applied loads^[^ ^109^ ^]^ (Figure 4a). To achieve full autonomy, the small amount of water can be supplied through autonomic perspiration, as demonstrated by Mishra et al.^[^ ^110^ ^]^ (Figure 4b). In their work, they developed a hydrogel‐based soft actuator capable of sweating to maintain a stable body temperature, which holds potential to supply water for assisting in self‐cleaning. Roy et al. have also developed fabrics that combine self‐cleaning and self‐healing capabilities^[^ ^111^ ^]^ (Figure 4c). They achieved self‐cleaning by designing superhydrophobic robotic surfaces, while their healing process is activated by heating. Smart fabrics are being used in various devices, e.g., assistive wearables, where they are mostly actuated or stiffened by embedded electrically driven fibers, e.g., shape‐memory wires.^[^ ^112^ ^]^ Therefore, future research could explore the potential of integrating self‐cleaning and self‐healing fabrics into robotics while designing actuation mechanisms with multifunctionality. For example, shape‐memory alloy (SMA) wires could be utilized both as actuators and as a heat source to activate the self‐healing process.

Fluidic‐driven soft robots have the potential for their actuation fluid to serve as a cleaning agent. For instance, in pneumatic‐driven soft robots, compressed air can be employed to dislodge contaminants from fracture surfaces. Similarly, in hydraulic‐driven soft robots, the actuation liquid can be utilized to rinse dirt particles from fracture surfaces that possess a (hyper)hydrophobic property.

## 4 Damage Closure

### 4.1 The Need for Damage Closure

Whereas biological systems exhibit remarkable capabilities for tissue growth and regeneration^[^ ^113^, ^114^ ^]^ and biohybrid robots^[^ ^115^, ^116^ ^]^ demonstrate regenerative potential when aided by the addition of external compounds,^[^ ^117^ ^]^ synthetic materials, in contrast, lack the ability to grow and regenerate.^[^ ^37^ ^]^ Consequently, it is essential to keep the fracture sides in close contact during the healing process.^[^ ^37^ ^]^ This recontact at the molecular level facilitates reactive compounds to reach the appropriate proximity for reforming the reversible bond across the fracture interface, thereby restoring the mechanical properties. In the human body, the proliferative phase of healing is responsible for filling the wound and regenerating new tissues.^[^ ^36^ ^]^ However, depending on the severity of the wound, external assistance such as stitching large lacerations may be necessary for proper healing.

Soft robots are commonly made from elastomers, such as silicone or rubbers.^[^ ^58^ ^]^ Their elastic recovery not only restores the soft robot to its initial state upon deactivation but also completely self‐closes small‐scale damages.^[^ ^19^, ^50^, ^118^ ^]^ Nevertheless, for large damages or damages occurring in specific locations, this elastic recovery alone is insufficient to seal the damage on a molecular level.^[^ ^37^ ^]^ In addition, to enable in situ and during operation self‐healing,^[^ ^119^ ^]^ keeping the damage actively closed becomes necessary.^[^ ^37^ ^]^ Apart from gravity that may reopen damages and impede the healing process in in situ self‐healing, actuation forces could also exacerbate damage or even result in the propagation of larger ones.

There are other scenarios where damage closure becomes necessary. The authors believe that the occurrence of large damages in a unclean environment requires swift damage closure to prevent particles from contaminating the fracture surfaces. In another instance, a sharp object or rupture may create rough surfaces at the site of the damage, impeding proper molecular‐level contact between the affected sides,^[^ ^34^ ^]^ despite the inherent elastic recovery of the self‐healing elastomer. Therefore, applying pressure to the sides enhances contact and would improve the healing efficiency. Furthermore, depending on the chemistry and mechanism of the healing process, self‐healing materials exhibit variability in the size of damage they can effectively repair, often influenced by the mobility of their polymer network structure.^[^ ^13^ ^]^ Although offering superior mechanical and thermal stability, less mobile networks typically require the application of an external pressing load during healing,^[^ ^120^ ^]^ a load that can be supplied by an integrated mechanism that is autonomously controlled. In another scenario, certain self‐healing materials necessitate triggering, such as heating or exposure to UV light.^[^ ^12^, ^13^ ^]^ Based on the author's experience, these stimuli may cause the material to shrink or expand during healing, and if not controlled, they may result in misalignment and damage opening. Therefore, actively maintaining gap closure will minimize these risks that lead to healing failure. Additionally, immediate closure of a potential gap in autonomous self‐healing materials is crucial to prevent aging effects.^[^ ^121^ ^]^

The active closure of damage can singularly shoulder the responsibility for healing, provided that the attractive forces can sustain the system's integrity. This implies that healing can occur even in materials not leveraging self‐healing chemistry. This principle aligns seamlessly with (liquid/ink‐based) magnetic composites,^[^ ^122^, ^123^ ^]^ which can autonomously or through the application of an external magnetic field seal the gaps and uphold the system's integrity overtime and under loads.

### 4.2 Technologies for Damage Closure

Following is a list of key damage closure mechanisms, including material‐based techniques to assistive robotic components (**Table** **2**). Generally, the gap is either filled with healing agents or closed using force, which may be provided by the release of stored energy in the material upon stimulation or by integrated assistive components.

**Table 2:** Comparison of different damage closure technologies in soft robotics.

Technology	Integration method	Damage size	Closure multiplicity	Activation	Demonstration	Healing efficiencya)	Figure	Ref
Extrinsic healing agents	Embedding supply tubes	Centimeter scale	One time	Pumping agents to solidify	Material specimens	63% in impac*t* test	Figure 5a	[127]
	Healing electrofluid actuators	Millimeter scale	Multiple times	By the damage	Soft manipulator	Robot function is mostly recovered	Figure 5b	[48]
Shape memory polymer materials	Property of the material	Microscale	Depends on reprogramming	Thermal	Material specimens	Up to 90% in tensile test	Figure 5d	[136]
SMA wires	Embedding SMA wires as reinforcement	Millimeter scale	Multiple timesb)	Thermal	SMA‐reinforced bending actuator	Actuator function is mostly recovered	Figure 5e	[37]
Magnetically closure	Composite material	Millimeter scale	Multiple times	By external magnetic field	Material specimens	Varies from 56% to 100%	Figure 5f	[154]
	Screen printing magnetic ink	Centimeter scale (on the fly)	Multiple times	By the damage	Swimmer in a petri dish	Varies from 53% to 88%	Figure 5g	[123]
Robotic actuation	McKibben muscles	Centimeter scale	Multiple timesb)	Pneumatic	Crawling robot	Robot function is mostly recovered	Figure 5h	[53]

a) The healing efficiency is not only dependent on the degree of damage closure but also on the material's healing property.

b) Multiple instances of damage closure were demonstrated, provided the SMA wires or McKibben muscles remained intact.

#### 4.2.1 Extrinsic Healing Agents

Healing with extrinsic healing agents can be viewed as a method of externally introducing material regeneration into synthetic self‐healing materials. Extrinsic self‐healing materials typically refer to those materials where the healing originates from additional substances (healing agents) embedded within the material via micro‐ or nano‐capsules or in hollow fibers or a vascular system.^[^ ^12^, ^13^ ^]^ Compared to intrinsic self‐healing materials, where healing relies on reversible chemical or physical bonds and requires microscopic closure of the damage,^[^ ^12^, ^13^ ^]^ extrinsic self‐healing materials are less constrained by the need for precise physical alignment during the healing process.^[^ ^13^ ^]^ Hence, extrinsic mechanisms are superior for self‐healing in stiff and brittle materials, where recontact is more challenging. Nevertheless, in typical extrinsic self‐healing materials, damage filling and healing are still limited to small‐scale sizes and a finite number of healing cycles due to the restricted amount of incorporated healing agents, which is more highlighted in capsule‐based extrinsic healing.^[^ ^12^, ^13^ ^]^ Due to design and processing challenges associated with embedding healing agents in soft elastomers,^[^ ^12^ ^]^ most of the developed extrinsic self‐healing materials are limited to stiff and brittle polymers (>100 MPa)^[^ ^124^, ^125^, ^126^ ^]^ and do not cover the typical range of softness (Young's modulus of 0.1–10 MPa^[^ ^11^ ^]^) required in the majority of soft robotics applications.

**Figure 5:**
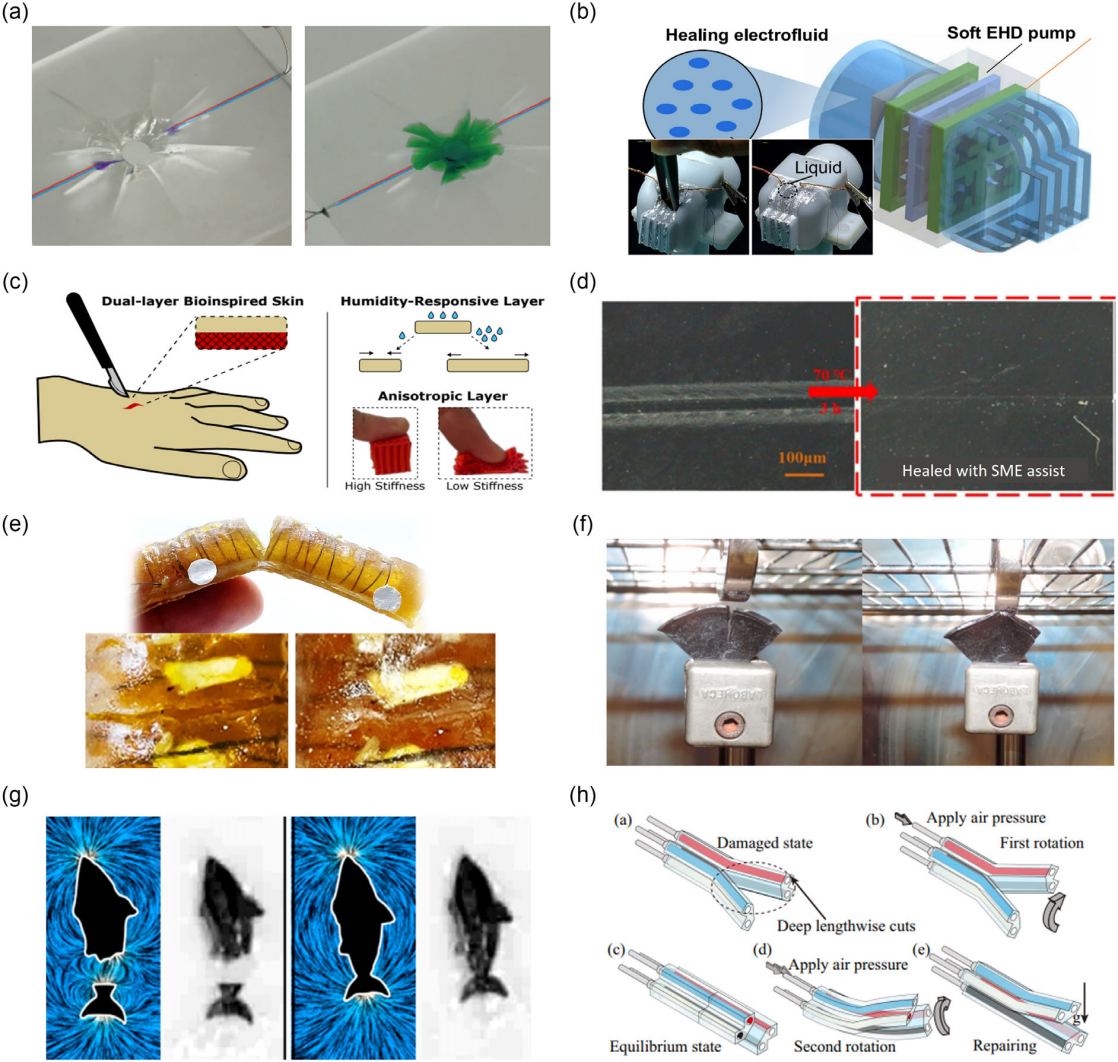
Technologies for damage closure. a) Integrating two microchannels delivering two externally provided healing agents. Reproduced with permission.^[^ ^127^ ^]^ Copyright 2020, AAAC. b) Healing electrofluid for actuation and also as healing agent. Reproduced (Adapted) with permission.^[^ ^48^ ^]^ Copyright 2023, The Authors, Published by Springer Nature. c) Controllable wound closure in hydrogels, by Hardman and Iida. Reproduced with permission.^[^ ^131^ ^]^ Copyright 2024, IEEE. d) Closing scratch using shape‐memory polymers. Reproduced with permission.^[^ ^139^ ^]^ Copyright 2018, American Chemical Society. e) Damage closure in bending actuators using SMA wires. Reproduced (Adapted) with permission.^[^ ^37^ ^]^ Copyright 2023, The Authors, Published by Springer Nature. f) Magnetically assisted damage closure. Reproduced with permission.^[^ ^154^ ^]^ Copyright 2020, Elsevier Ltd. g) Healing on the move assisted by magnetic forces. Reproduced with permission.^[^ ^123^ ^]^ Copyright 2021, American Chemical Society. h) A crawling soft robot powered by McKibben muscles that can also close damages. Reproduced with permission.^[^ ^53^ ^]^ Copyright 2024, The Authors, Published by IEEE.

Nevertheless, healing agents can be externally supplied and do not be embedded within the polymer matrix.^[^ ^32^, ^48^, ^96^, ^119^, ^127^, ^128^ ^]^ White et al. demonstrated the ability to fill and heal large damage sizes, with a diameter of up to 35 mm, with an efficiency of 62 % derived via impac*t* testing. They achieved this by integrating two microchannels delivering two externally provided healing agents that fill the damage and polymerize in two stages^[^ ^127^ ^]^ (**Figure** **5**a). However, the constraint on multiple damage‐healing cycles remains valid since the microchannels are embedded within the material. Furthermore, their healing is limited to the locations where the channels are integrated. Matsuda et al. developed a double‐network hydrogel comprising soft and brittle networks.^[^ ^96^ ^]^ Upon damage, the breakage of the brittle network generates mechanoradicals, which form new networks through the external supply of monomers, resulting in growth in strength and size.^[^ ^96^ ^]^ Additionally, the use of healing agents is being explored in biological materials. For instance, Roman et al. demonstrated a biohybrid robot that heals with the assistance of a biological glue incorporating myoblast cells that give rise to the growth of muscle cells.^[^ ^117^ ^]^ In another scenario, the addition of water can serve as an agent for damage closure through the swelling effect in hydrophilic self‐healing materials.^[^ ^129^ ^]^ In particular in self‐healing hydrogels, swelling occurs due to changes in humidity and in pH, which can assist in damage closure and healing.^[^ ^130^ ^]^ Aside from material composition, the morphological design of composites or dual‐layer skins can lead to more control over both closing and opening of damages, as illustrated by Hardman et al.^[^ ^131^ ^]^ (Figure 5c). Consequently, controlled anisotropic wound closure can be achieved, optimizing the closure based on anticipated damage and actuation deformations.

Fluid‐driven mechanisms are commonly used for actuation in soft robotics. Given that healing agents are typically embedded in a liquid phase, there is an opportunity to repurpose the actuation medium for healing. Thus, the fluid performs a dual, multifunctional role, acting both as the actuation fluid and the healing agent, as shown in the work of Wallin et al. who used a fast curing resin as actuation fluid.^[^ ^32^ ^]^ Upon damage, the fluid is exposed to air and sunlight and polymerizes, thereby sealing and healing the damage. Recently, healing electrofluids have also been utilized as both the actuation medium and the healing agent.^[^ ^48^, ^128^ ^]^ These liquids solidify upon exposure to the air, forming a physical bond with the membrane chamber of the robot at the damaged site. They have recently found application in soft fluidic robots driven by soft electrohydrodynamic pumps^[^ ^48^, ^128^ ^]^ (Figure 5b). Additionally, the inherent healing ability of dielectric liquids can be exploited to address electrical breakdowns in dielectric fluid actuators, such as hydraulically amplified, self‐healing, and electrostatic actuators.^[^ ^132^, ^133^ ^]^ The recent work of Li et al. shows a universal gripper wherein the membrane is filled with different liquids to boost the robot's properties, such as damage resilience via filling the membrane with a viscous glue.^[^ ^119^ ^]^ The advantage of liquids lies in their ability to penetrate damage gap and seal it, effectively addressing physical barriers in material healing. However, the risk of leaking should be taken into account, especially in cases of extensive damage or in positions where gravity exacerbates the leakage.

In summary, while most recently developed self‐healing soft robots leverage intrinsic self‐healing materials,^[^ ^12^, ^20^, ^21^, ^22^ ^]^ the author anticipates a growing trend of robots utilizing extrinsic healing agents in the near future. This shift will be driven by the acknowledgment that physical barriers present substantial challenges in the healing of intrinsic self‐healing polymers.

#### 4.2.2 Shape‐Memory Polymers

Shape‐memory effect (SME) is a common property found in many polymers and is being widely researched for different soft robotic applications, e.g., manipulation and locomotion.^[^ ^22^, ^69^, ^120^, ^134^, ^135^ ^]^ The most prevalent type of SME is the heat‐activated one‐way SME, where the material is mechanically deformed from its permanent shape into a temporary programmed shape. This programming usually involves heating the material above its (glass) transition temperature, applying stress to change its shape, and then cooling down it below the transition temperature to fix the temporary shape. Upon reheating above its transition temperature, the material recovers its permanent shape. Researchers have used this principle for closing microcracks^[^ ^136^, ^137^, ^138^, ^139^ ^]^ (Figure 5d). Ahmed et al. demonstrated that magnetically induced heat can restore the permanent shape of a self‐healing composite with embedded magnetic nanoparticles after being significantly deformed by necking during a tensile test.^[^ ^97^ ^]^ After necking, inductive heating triggers the shape‐memory response, aligning the corresponding fracture surfaces with excellent contact, thereby enhancing the healing process. Demonstrations of shape‐memory‐assisted healing typically involve applying a pre‐stretch as a temporary shape (with the damage itself potentially causing the stretch in the material^[^ ^139^ ^]^), the occurrence of damage, and then triggering the material to recover its permanent shape and close the crack. However, most studies remain confined to the material level due to several factors: 1) the ability to fix the temporary shape of a material necessitates a glass transition temperature that exceeds room temperature. This means that the material does not possess the necessary softness for applications in soft robotics.^[^ ^11^ ^]^ 2) The application of an external load is required for shape programming, otherwise damage closure is only possible for one cycle. This challenge can be addressed in the robotics systems via exploiting the actuation force,^[^ ^140^ ^]^ or implementing an antagonistic design.^[^ ^141^ ^]^ 3) Only minor cracks can be closed, as larger damages release the stored stress in the material and may fully diminish the shape recovery property. Zhang et al. employed a composite approach wherein they integrated shape‐memory muscles (e.g., polyethylene polymeric helical springs) into a self‐healing matrix.^[^ ^142^ ^]^ While this approach can address damages on the millimeter scale, it is constrained to damages that do not impact the non‐self‐healing polyethylene muscles.

As opposed to the one‐way SME, there exist two‐way SME where the material reversibly changes when the stimulus is turned on or off, e.g., heated above or cooled down below the transition temperature.^[^ ^143^ ^]^ This ability is potentially interesting for autonomously closing microcracks in multiple times, despite the challenges that exist in programming and controlling the two‐way shape memories.^[^ ^134^ ^]^ It is also important to note that the two‐way SME property is primarily found in crystalline or semicrystalline polymeric networks.^[^ ^134^ ^]^

#### 4.2.3 SMAs

The SME is also observed in few alloys (e.g., nickel–titanium) where the transition between shapes arises from a change in the crystalline structure of the metal, specifically transitioning from martensite to austenite.^[^ ^144^ ^]^ In these SMAs, the stress‐induced phase transformation that forms a temporary shape is reversed upon heating the alloy. As such, temporary shape programming depends only on the applied stress while the recovery is based on the temperature threshold transition. In the past decade, SMA‐assisted healing has been studied in simple specimens where the integrated pre‐stretched one‐way SMA wires in the soft matter are contracted upon Joule‐heating and push the damage sides against each other to close the damage.^[^ ^125^, ^145^, ^146^, ^147^, ^148^, ^149^ ^]^ Based on the length of the SMA wires and the applied pre‐strain (usually 5–8% of the length of the wire), different damage sizes can be closed. In comparison to shape‐memory polymers, SMA wires have the potential to close larger gaps due to their greater stroke. This stroke can be further amplified when SMA wires are configured in a coil, albeit at the expense of exerting less load. Similar to shape‐memory polymers, there are two‐way SMAs which can memorize two different shapes and transit between them by just the change in temperature.^[^ ^144^, ^150^ ^]^ However, due to their complex function and cyclic degradation,^[^ ^151^ ^]^ they are less prevalent and have received less attention in robotic applications and damage closure.

Recently, Kashef Tabrizian et al. developed a new class of fiber‐reinforced soft pneumatic actuators, where the conventional soft materials are replaced by self‐healing polymers and traditional reinforcements are substituted by SMA wires.^[^ ^37^ ^]^ This innovation allows the actuator to autonomously heal wide‐opened cracks in the millimeter scale (Figure 5e). Upon electrical activation, the SMA wires heat up through the Joule effect, contracting the soft chamber to close the damage while simultaneously heating the material. This enables and accelerates the healing process. Despite working with one‐way SMA wires, Kashef Tabrizian et al. have illustrated that multiple damage–closure–healing cycles can be achieved without external assistance through careful design, in their case by embedding helical SMA wires and utilizing the elastic response of the self‐healing polymer.^[^ ^37^ ^]^

This recent advancement in using SMA wires as healing‐assistive mechanisms, along with their development as actuators for soft robotics, opens new possibilities for utilizing SMA wires as versatile tools in this field. For instance, Kashef Tabrizian et al. have demonstrated that with minor adjustments to their work on SMA wires for damage closure,^[^ ^37^ ^]^ these wires can be employed in a hybrid pneumatic‐electric actuation setup, enabling bending and twisting motions in SMA‐wire‐reinforced soft actuators.^[^ ^152^ ^]^

#### 4.2.4 Magnetically Closure

The attraction force that magnetic materials exert on each other is potentially interesting to be exploited to retain the lost integrity in the soft matters. Recently, there has been a detailed review of self‐healing magnetic composites, which are created by incorporating magnetic nanoparticles into soft elastomer matrices.^[^ ^153^ ^]^ Additionally, there has been a review of self‐healing magnetic actuators,^[^ ^22^ ^]^ as well as a broader examination of magnetically responsive actuators.^[^ ^69^, ^135^ ^]^ The recovered integrity of the material due to magnetic forces can either heal the function of the system (even if the material itself is not intrinsic self‐healing)^[^ ^74^, ^122^, ^123^ ^]^ or assist with the self‐healing by closing damage and creating molecular recontact that enables rebinding across the fracture surface^[^ ^154^, ^155^ ^]^ (Figure 5f). When aiming to develop soft robots capable of autonomously recovering from damage through self‐closure, permanent magnetic self‐healing polymers, which utilize ferromagnetic fillers, offer advantages,^[^ ^122^ ^]^ compared to paramagnetic self‐healing polymers. This is because paramagnetic polymers require an external magnetic field for their magnetization.^[^ ^74^, ^154^, ^156^ ^]^ This increases the system's complexity, and furthermore, applying an external magnetic field may induce unintended deformations in the soft robotic body^[^ ^154^ ^]^ (Figure 5f).

Karshalev et al. created a miniature swimmer robot equipped with permanent magnetic strips. Upon being cut in half, the strip transforms into two separate permanent magnet strips that attract each other, autonomously restoring the robot's structural integrity and functionality, even during swimming^[^ ^123^ ^]^ (Figure 5g). With the recent surge in research dedicated to magnetically activated robots, the potential for magnetic‐assistive self‐healing in soft robotics is significantly increasing, exemplified by the work of Cheng et al.^[^ ^157^ ^]^

#### 4.2.5 Closure upon Robotic Actuation

Soft robots are powered by various actuation principles, including fluidic‐driven, tendon‐driven, dielectric elastomer, and SMA‐driven actuators.^[^ ^58^, ^158^, ^159^ ^]^ These integrated actuators can not only be used to move the robot but can also be controlled to close potential gaping damages in the soft robotic body. Of course, the damage must not be so severe as to render the robot nonfunctional. Following this principle, Xie et al. developed a crawling soft robot actuated by four McKibben muscles embedded within a self‐healing structure.^[^ ^53^ ^]^ Upon damage, the contraction of the muscles closes the gap of up to 1.6 mm, even if the material is removed. Furthermore, lengthwise cuts could be closed and partially aligned by position reorientation so that gravitational force aids in closing the gap, i.e., using the weight of the robot for damage closure^[^ ^53^ ^]^ (Figure 5h).

In many of the developed SMA‐wire/tendon‐driven soft actuators, the wires/tendons are asymmetrically embedded in the soft material to enable actions like bending or torsion motions.^[^ ^160^, ^161^, ^162^, ^163^ ^]^ However, by integrating wires/tendons symmetrically relative to the neutral axis, it becomes possible to simultaneously activate the wires, leading to a lengthwise contraction of the soft material and the closure of potential cuts or gaps perpendicular to the length direction of the actuator. Additionally, a bending motion can also close gaps on the side of the actuator that is located on the inner curve of the bend. This principle also applies to fluidic‐driven actuators, assuming that the damage is not too deep to result in fluid loss.^[^ ^53^ ^]^ In pneumatic bending actuators, applying vacuum instead of inflation can sometimes change the bending direction, which can provide opportunities to close more damages. In general, these principles can be applied to other types of actuation in soft robotics.

## 5 Material Healing

It is worth mentioning that this phase of the self‐healing process of soft robots has been the main focus of the previously published review papers,^[^ ^12^, ^23^, ^54^, ^164^ ^]^ which delve into the chemistry of different types of self‐healing materials and, for the nonautonomous ones, their corresponding healing stimuli. Here, the emphasis is not on the chemistry of the materials, but rather on how the required stimulus, if necessary, can be supplied by the robotic system itself, making the process autonomous on the system level.

### 5.1 The Need for Healing Stimuli

While the application of a stimulus, such as heat, pH, or light, enables self‐healing in nonautonomous self‐healing polymers,^[^ ^3^, ^12^, ^14^, ^15^, ^16^ ^]^ it in many cases accelerates the healing process of autonomous self‐healing polymers, which are also capable of self‐healing at ambient conditions, yet at a slower rate. For instance, raising the temperature of most self‐healing polymers enhances the mobility and reactivity of their polymeric network,^[^ ^52^, ^121^ ^]^ or the embedded healing agent,^[^ ^48^, ^125^ ^]^ ultimately leading to more effective and faster healing. Enhancing the mobility of the polymeric network can also assist in closing the damage at the microscopic level, a phenomenon referred to as the “zipping effect” as illustrated by Terryn et al.^[^ ^50^ ^]^

Autonomous healing in intrinsic self‐healing polymers is primarily effective when the damage is immediately or shortly closed after its occurrence. If not, the reactive groups resulting from the mechanical breakage of reversible bonds begin to react and reform the bonds separately on the fracture surfaces, depleting the healing capacity.^[^ ^50^, ^121^ ^]^ Overtime, if the two damaged sides are not reconnected, the individual parts reach an equilibrium state where almost all bonds are reformed and reactivity is low, a phenomenon described as “aging” by Diaz et al.^[^ ^121^ ^]^ In that case, a stimulus, such as heat or light, can reactivate the healing capacity by thermally or photochemically breaking the reversible bond at the fracture surfaces. Another issue with autonomous self‐healing materials arises when the fracture surfaces are not properly aligned, leading to misalignments (e.g., ugly scars). Since self‐healing begins autonomously whenever the fracture surfaces recontact, correcting these misalignments is often challenging, if not impossible. This is akin to situations where a poorly healed broken limb in the human body needs to be deliberately re‐broken to allow for proper healing.

Although using a stimulus to initiate and/or accelerate the healing process of the self‐healing materials adds complexity to the system, it offers greater control over the healing action, allowing for on‐demand healing.^[^ ^29^ ^]^ When using nonautonomous self‐healing polymers alongside an integrated stimulus‐providing system, it becomes possible to initiate healing precisely when and where it is needed. This is advantageous because, in some cases, the healing process needs to be postponed, allowing time to clean, realign, and close fracture surfaces.

Soft robotic applications are diverse, from robots made of ultrasoft materials^[^ ^46^ ^]^ to more stiff ones.^[^ ^3^ ^]^ As such, to introduce self‐healing to a wide range of applications, self‐healing polymers with different properties are required. Generally, autonomous self‐healing polymers have a low Young's modulus (typically below 1 MPa).^[^ ^26^, ^50^ ^]^ In contrast, stiffer and stronger self‐healing polymers, which can be used to create soft robots capable of exerting higher loads, usually require a stimulus for self‐healing.^[^ ^12^ ^]^ Nonetheless, material‐based research is pushing the boundaries of autonomous self‐healing polymers, striving to break the existing trade‐off between mechanical properties and autonomous self‐healing, as well as the healing time.^[^ ^165^, ^166^ ^]^

Overall, the necessity for a stimulus‐providing system is in many cases evident, whether to enable or accelerate the self‐healing process.

### 5.2 Technologies for Healing Stimuli

Next to the thermal‐, light‐, and water‐triggered self‐healing technologies discussed in this section, it is important to mention the presence of pH‐responsive self‐healing materials. However, pH‐activated self‐healing materials are not addressed here due to their restricted applicability beyond certain biomedical and chemical contexts, primarily due to the limited control over pH. pH responsiveness is typically observed in hydrogels, which expand in acidic conditions and contract in basic environments.^[^ ^130^ ^]^ These pH‐responsive hydrogels are extensively researched for applications in in‐body drug delivery.^[^ ^167^ ^]^

#### 5.2.1 Joule‐Effect Heating

**Figure 6:**
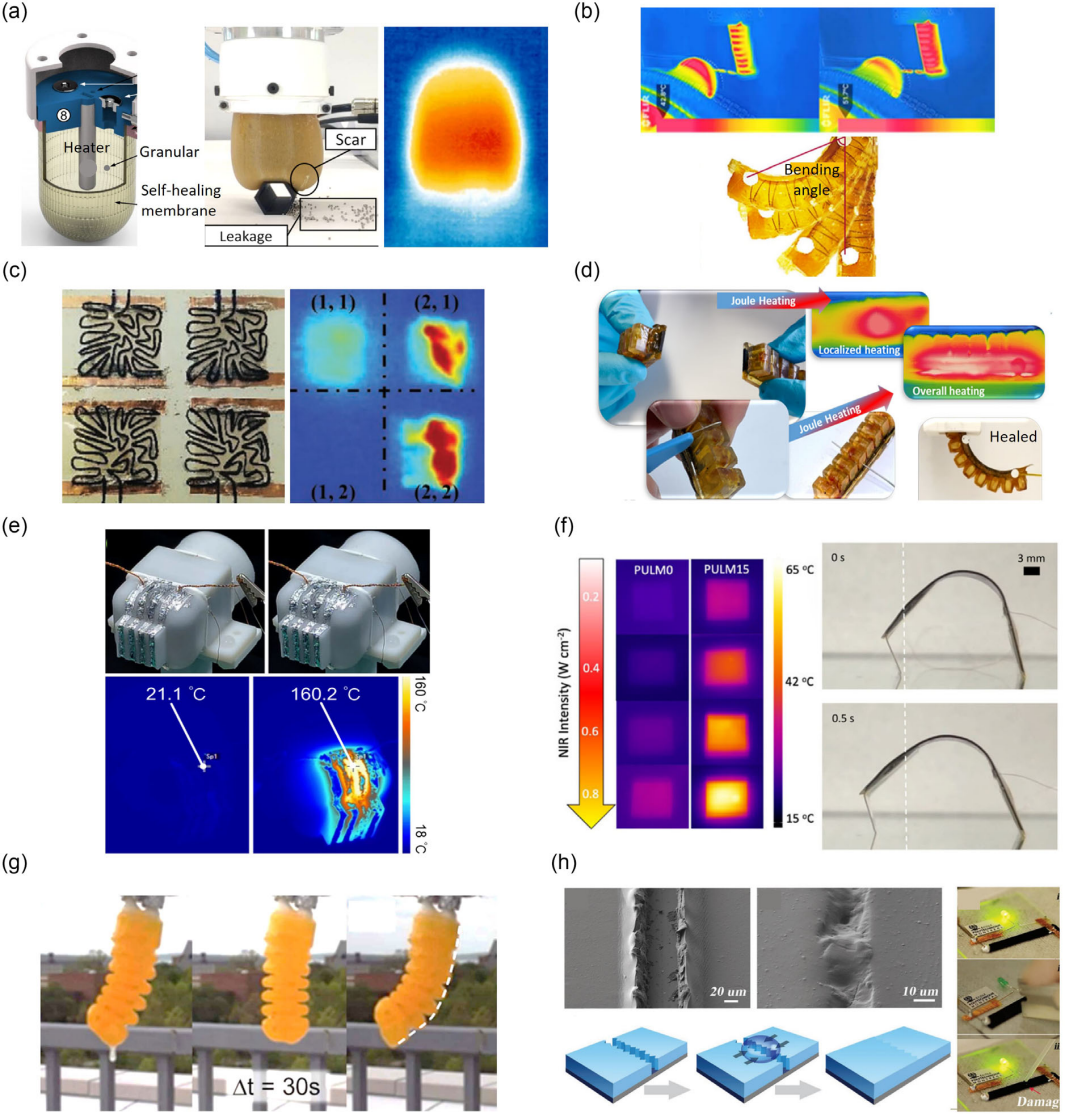
Stimuli providing systems for self‐healing robotics. a) Commercial Joule‐effect heater integrated inside the self‐healing universal‐jamming gripper. Reproduced with permission.^[^ ^49^ ^]^ Copyright 2023, The Authors, Published by Wiley‐VCH GmbH. b) Integrated SMA wires heat the bending actuator up by Joule effect. Reproduced (Adapted) with permission.^[^ ^37^ ^]^ Copyright 2023, The Authors, Published by Springer Nature. c) Multifunctional self‐healing electronics skin with the Joule‐effect heating layer. Reproduced (Adapted) with permission.^[^ ^55^ ^]^ Copyright 2020, WILEY‐VCH GmbH. d) Embedded self‐healing Joule‐effect heater in the self‐healing bending actuator. Reproduced (Adapted) with permission.^[^ ^29^ ^]^ Copyright 2022. IEEE. e) Joule‐effect liquid metal heating skin to accelerate curing of the healing electrofluid. Reproduced (Adapted) with permission.^[^ ^48^ ^]^ Copyright 2023, The Authors, Published by Springer Nature. f) Heating by photothermal effect in the dielectric elastomer actuator. Reproduced with permission.^[^ ^196^ ^]^ Copyright 2022, The Authors, Published by Springer Nature. g) Curing of the healing agent under sunlight in the soft actuator. Reproduced with permission.^[^ ^32^ ^]^ Copyright 2017. The Royal Society of Chemistry. h) Healing by 10 μL drop of deionized water in the conductive film. Reproduced with permission.^[^ ^215^ ^]^ Copyright 2017. WILEY‐VCH GmbH.

Joule‐effect heating occurs when electrical current passes through a material, generating heat and causing a rise in temperature. Traditional electrical heaters, like heat cartridges, utilize this principle and can be strategically positioned in areas of the soft robotic body that do not deform upon actuation^[^ ^49^ ^]^ (**Figure** **6**a). Nevertheless, the bulkiness and/or rigidity of these heaters impede their embedding in many self‐healing soft robots without compromising their inherent softness. Hence, flexible heaters, such as conductive wires^[^ ^37^, ^83^, ^168^ ^]^ (Figure 6b) and conductive fabrics,^[^ ^169^ ^]^ have been integrated into self‐healing soft robots to stimulate healing without undermining the flexibility of the robotic system. However, similar to the soft robot in which they are embedded, these flexible heaters themselves can also be vulnerable to damage.

To address this issue, self‐healing (flexible) heaters have been under development (Figure 6c–e). The majority of self‐healing polymers are insulators due to the lack of free electrons needed for conductivity. Nevertheless, by dispersing conductive fillers within a self‐healing polymer matrix, such as carbon nanoparticles,^[^ ^29^ ^]^ carbon nanotubes,^[^ ^71^, ^170^, ^171^ ^]^ and Ag (“silver”) + NW (“nanowire”)s,^[^ ^55^, ^172^ ^]^ the self‐healing matrix becomes electrically conductive, while preserving its healing ability.^[^ ^82^ ^]^ Compounding with these fillers reduces the network mobility in the resulting composites, decreasing their healing capacity. Therefore, optimizations on filler type, filler content, and dispersion technique to minimize the trade‐off between electrical conductivity and self‐healing capacity have been the topic of many research studies. In search of enhancing the self‐healing ability in composites, hybrid fillers, e.g., a combination of fillers, have been investigated. As illustrated by Sahraeeazartamar et al.^[^ ^173^ ^]^ the combination of carbon black and clay particles leads to new morphologies that allow to reduce the fill content and result in better healing performances. Although many have reported on self‐healing heaters, such as the biodegradable heater of Guo et al.^[^ ^174^ ^]^ and others collected in a review paper by Orellana et al.^[^ ^82^ ^]^ very few have been implemented in robotic demonstrators. Kashef Tabrizian et al. did embed a self‐healing heater in a self‐healing bending actuator, where the heater provides the temperature raise needed for healing of itself and also the other parts of the actuator^[^ ^29^ ^]^ (Figure 6d). Tang et al. have coated a liquid metal–based electronic skin on top of the body of their actuator, driven by liquid electrofluids. The electronic skin can heat up to 160 °C and accelerates the curing of the healing electrofluids^[^ ^48^ ^]^ (Figure 6e).

Joule‐effect (self‐healing) heaters demonstrate physical intelligence, a concept described by Metin Sitti in his review paper.^[^ ^175^ ^]^ When damage occurs and the material recontacts, the resistance locally increases at the damaged site.^[^ ^29^ ^]^ Consequently, when current is applied, heating becomes concentrated at this location, precisely where it is needed most (Figure 6d). This phenomenon accelerates the heating process, contributing to enhanced energy efficiency.^[^ ^29^, ^71^ ^]^ Moreover, localized heating can prevent the heating of other components in a system that would be sensitive to overheating. Aside from this self‐targeted heating, many conductive polymer composites possess a built‐in self‐regulating mechanism that acts as a natural temperature limiter, effectively preventing the system from reaching excessively high temperatures.^[^ ^176^ ^]^ This property, known as the positive temperature coefficient effect, causes the resistance to increase with rising temperature.^[^ ^29^, ^177^ ^]^ Such temperature sensitivity also enables self‐sensing of the temperature.^[^ ^29^ ^]^

Aside from self‐healing, Joule‐effect heaters have been extensively utilized in wearable healthcare devices,^[^ ^178^, ^179^, ^180^ ^]^ variable stiffness structures,^[^ ^168^, ^181^ ^]^ and generally as electrothermal actuators.^[^ ^69^, ^169^, ^182^, ^183^, ^184^ ^]^ Therefore, the authors envision a future in which Joule‐effect heaters embedded in soft robots can serve a multipurpose, promoting self‐healing while simultaneously providing actuation or stiffness modulation. Nevertheless, it is crucial to determine whether healing can occur simultaneously with these other functionalities, as is presented by Tonazzini et al.^[^ ^168^ ^]^ If not, there should be a distinction between the healing temperature and the actuation temperature.

#### 5.2.2 Induction Heating

Induction heating is a process that employs an alternating magnetic field to heat an electrically conductive or magnetic material.^[^ ^185^, ^186^ ^]^ In electrically conductive materials, an electrical current (eddy current) is induced when the material is exposed to a changing magnetic field, generating heat via the Joule effect.^[^ ^186^ ^]^ Additionally, heat can be produced through hysteresis heating in ferromagnetic materials and relaxation losses in superparamagnetic materials.^[^ ^186^ ^]^ Induction heating provides a contactless heating method, eliminating the need to connect electrodes to soft materials. However, it requires a system to generate the alternating magnetic field. By using induction heating, conductive or magnetic materials encapsulated within nonconductive materials can be wirelessly and locally heated.^[^ ^186^, ^187^ ^]^ The authors believe that induction heating holds potential for localized heating in case of damage.

While existing research explores triggering self‐healing in polymers via induction heating,^[^ ^164^, ^186^, ^187^, ^188^, ^189^, ^190^ ^]^ to the authors’ knowledge, this approach has not yet been integrated into soft robotics. Nevertheless, induction heating has found utility in various other functions within soft robotics, including the development of bending sensors,^[^ ^191^ ^]^ and the creation of wireless heaters to actuate heat‐activated untethered robots.^[^ ^192^ ^]^ This highlights the potential to utilize induction heating for healing purposes, serving as a stimuli provider for healing, as well as for the primary functions of robots, such as crawling.^[^ ^192^ ^]^

#### 5.2.3 Photothermal Effect

The photothermal effect in polymers is based on compounds with a strong light absorption across a broad spectrum, which can be either specific fillers incorporated into the polymer matrix, including carbon‐, metal‐, metal‐oxide‐, or ligand‐based particles,^[^ ^193^, ^194^ ^]^ or specific polymer backbones, such as polydopamine.^[^ ^195^ ^]^ When exposed to light, these light‐absorbing compounds convert the radiant energy into heat. In self‐healing polymers, this thermal energy can be harnessed to disrupt the thermoreversible cross‐links within the polymer structure, thereby enhancing molecular mobility and dynamic interactions, enabling or accelerating the self‐healing. Upon cooling, such as when the irradiation is terminated, the thermoreversible bonds rebind, restoring the original material properties both in bulk and across any fracture surfaces that may exist.^[^ ^164^, ^194^ ^]^ The healing process can be controlled by adjusting the intensity, wavelength, and duration of the irradiation, enabling targeted healing.^[^ ^196^ ^]^ Through the use of point sources, such as lasers or LEDs, the irradiation can be concentrated onto a specific surface, enabling localized healing. Furthermore, the photothermal approach does not necessitate physical contact, allowing to control the self‐healing from a distance.^[^ ^194^ ^]^ Yet, this method encounters challenges due to the limited penetration depth of light, restricting self‐healing to surface‐level damages.^[^ ^12^, ^194^ ^]^ While increasing the light intensity can extend the penetration depth to some degree, it may also induce thermal degradation at the material's surface. Nevertheless, augmenting the thermal conductivity of the self‐healing polymer, such as by incorporating boron nitride or aluminum nitride, presents another avenue to facilitate heat conduction deeper into the material.

Apart from self‐healing applications, the photothermal effect is widely investigated to achieve other functionalities in soft robotics. For instance, it is utilized to induce mechanical motion in heat‐activated soft robots by leveraging on anisotropic thermal expansion property within the robot structure.^[^ ^135^, ^197^, ^198^ ^]^ Additionally, it finds various biomedical applications.^[^ ^199^, ^200^, ^201^ ^]^ Hence, there is potential to employ the photothermal effect for various functions, such as integrating healing activation with actuation or modulating the properties of dielectric elastomer actuators^[^ ^196^ ^]^ (Figure 6f) or it can be utilized to combine healing activation with the creation of light‐sensitive sensors.^[^ ^202^ ^]^ Furthermore, the photothermal effect can simultaneously serve for both healing and shape‐memory activation.^[^ ^22^, ^203^ ^]^ If there is a desire to decouple the healing action from these other functions of the soft robot, selectivity can potentially be created by using multiple light‐absorbing fillers that are specific to divergent wavelengths^[^ ^196^ ^]^ or by varying the polarization of the light source.^[^ ^198^ ^]^ Additionally, Liu et al. have demonstrated how the photothermal effect can facilitate 3D assembly in soft robots and enable modularization and multifunctionality through photo‐welding.^[^ ^204^ ^]^ It is worth highlighting that sunlight can be harnessed as the energy source for photothermal activation, demonstrated for both healing activation,^[^ ^194^, ^203^ ^]^ and also for motion activation in robots.^[^ ^205^, ^206^ ^]^

#### 5.2.4 Photoinduced Healing

Photoinduced intrinsic self‐healing typically involves a photoreversible reaction,^[^ ^194^, ^207^ ^]^ such as the anthracene^[^ ^208^ ^]^ coumarin cycloaddition reaction^[^ ^209^ ^]^ and the photoinduced metathesis of diselenide.^[^ ^210^ ^]^ This so‐called photoreversible self‐healing typically involves irradiating the polymer with one or more lights with different wavelengths, depending on the mechanism of the reversible reaction involved in the healing process. Both coumarin and anthracene, for instance, undergo photodimerization when subjected to wavelengths above 300 nm, which can be utilized to cross‐link photoreversible self‐healing polymers.^[^ ^208^, ^209^ ^]^ When exposed to light with wavelengths below 300 nm, it results in the reversible cleavage of these cross‐links. Consequently, damages can be healed by subjecting these polymers to a sequence of light wavelengths, first below and then above 300 nm. Depending on the specific chemistry or network composition, visible light alone is sufficient for reestablishing the cross‐links, simplifying the integration of this healing method in applications such as soft robots, as only one controlled emitter in the UV spectrum is required, rather than two. Nevertheless, to the best of the author's knowledge, no integrated emitting device for healing has yet been demonstrated in soft robots. But, using sunlight can be an automated mechanism for the healing activation.^[^ ^32^, ^211^, ^212^ ^]^ Stimuli‐driven self‐healing polymeric actuators, including light‐driven ones, have been recently reviewed.^[^ ^22^, ^24^ ^]^ Like photothermally triggered self‐healing, photo‐initiated self‐healing encounters challenges related to the restricted penetration depth of light. Nevertheless, a recent study demonstrates remarkable self‐healing of damage up to a centimeter below the surface by utilizing photo‐reversible dithiocarbamate bonds covalently linked to indole chromophores and benzyl groups.^[^ ^211^ ^]^

Alternatively, extrinsic photo‐initiated self‐healing mechanisms, which depend on a healing agent in the form of a photo‐curable resin, have been devised and integrated into soft robots. This method is exemplified by Wallin et al.,^[^ ^32^ ^]^ who introduced a hydraulic soft robotic bending actuator with a photocurable resin as its actuating fluid. Upon sustaining damage, the resin leaks out and encounters UV irradiation, leading to its polymerization and subsequent healing of the damage (Figure 6g).

#### 5.2.5 Water‐Triggered Healing

Water is another stimulus in initiating and expediting the healing process in some self‐healing materials, such as those containing hydrogen bonds,^[^ ^31^, ^165^, ^213^, ^214^ ^]^ ester bonds,^[^ ^214^ ^]^ and imine bonds.^[^ ^165^, ^166^ ^]^ Just like mechanisms that involve heat assistance, water can induce the breaking of chemical bonds, increasing the mobility of the network.^[^ ^31^, ^166^, ^214^ ^]^ Subsequent drying leads to the reformation of these bonds, facilitating the healing of the material.^[^ ^166^ ^]^ Water can also cause swelling, leading to an increase in softness and mobility of the polymer or hydrogel network and is therefore useful for healing^[^ ^129^, ^215^ ^]^ (Figure 6h). Additionally, this method can facilitate a close‐then‐heal approach, combining self‐sealing and self‐healing processes triggered by contact with water. It is important to note that swelling typically occurs in hydrophilic materials. However, many hydrophilic self‐healing polymers are not inherently stable in the presence of water. Prolonged exposure can cause extensive breaking of reversible bonds, leading to the dissolution or degradation of the polymer network. To address this challenge, a combination of hydrophilic and hydrophobic components can be employed within the material's network.^[^ ^165^ ^]^ In this case, the hydrophilic components are responsible for the swelling effect, which aids in healing, while hydrophobic components serve as stabilizers, preventing excessive degradation caused by prolonged water exposure. Alternatively, a double network structure,^[^ ^216^ ^]^ consisting of an irreversible network, can offer mechanical stability even at high water content or humidity.

Robots operating in outdoor environments could be equipped with sufficient intelligence to perform self‐repair by utilizing water readily available in nature or by taking advantage of precipitation.^[^ ^164^ ^]^ There have been reports on underwater robots,^[^ ^44^, ^217^, ^218^, ^219^ ^]^ as well as electronic skins designed for underwater applications.^[^ ^165^, ^215^ ^]^ However, integrating water‐enabled self‐healing materials for underwater applications requires careful consideration. Water‐responsive self‐healing materials may undergo changes in their mechanical properties when exposed to water. Therefore, the environment in which healing is practiced cannot always be the same environment in which the robot operates. To address this challenge, researchers have explored the use of ion–dipole interactions, which involve compounds consisting of a polymer with numerous dipoles and hydrophobic ionic liquids, to develop underwater self‐healing materials,^[^ ^220^ ^]^ as well as actuators.^[^ ^221^ ^]^ Zhang et al. reported on a conducting film that remains nonresponsive to external damage when wet, ensuring the continuous operation of electronic skin in wet conditions.^[^ ^215^ ^]^ Moreover, such materials can be integrated into water‐actuated soft robots,^[^ ^222^ ^]^ enabling the actuation stimuli to also serve as healing stimuli. This form of healing can also be considered as preventive healing.

## 6 Healing Assessment

### 6.1 The Need for Healing Assessment

Healing can be hindered by several factors, including but not limited to insufficient recontact, misalignment, and contamination, potentially leading to unsuccessful outcomes.^[^ ^34^ ^]^ Consequently, in real applications, healing may result in only partial recovery or could fail entirely. Identifying partial or failed healing can potentially save time and costs, as returning to operation in an unhealthy condition might exacerbate the problem.

In the context of self‐healing soft robots, system recovery encompasses two distinct aspects: the restoration of material properties and the reestablishment of robot function. To restore the material mechanical properties, both physical and chemical recovery are necessary. In the case of conductive self‐healing materials, the restoration of conductivity after damage healing should also be considered. However, it should be noted that the restoration of conductivity primarily depends on physical recovery, such as precise damage closure and realignment. This recovery, however, is only maintained as long as the system remains at rest and unloaded. Nevertheless, in many cases, a complete restoration of material properties, such as strain and stress fracture properties might not be essential for reinstating the robot's functionality, although it might reduce the lifetime of the robot. In the work of Kashef Tabrizian et al., while the self‐healing efficiency of the materials are limited, the FinRay gripper recovered its function after damage healing cycles. However, the lifetime of the healed gripper is slightly lower than the pristine one.^[^ ^30^ ^]^ The recovery of a robot typically includes the restoration of mechanical performance or its main function (e.g., bending performance,^[^ ^19^ ^]^ load‐bearing capacity,^[^ ^30^ ^]^ crawling properties^[^ ^53^ ^]^), together with the recovery of the robot's supplementary functions such as sensory^[^ ^34^, ^61^ ^]^ or heating^[^ ^29^ ^]^ capabilities.

Healing assessment is essential not only for evaluating self‐healing efficiency,^[^ ^21^ ^]^ but also for determining whether the self‐healing process has altered the properties of the robotic system, enabling recalibration or compensatory mechanisms to be implemented if needed. For example, self‐healing sensors used for both damage detection and health monitoring might need to be recalibrated after healing. Apart from environmental effects and strain‐induced permanent changes, the healing stimuli can also alter the sensor's response.^[^ ^76^ ^]^ Healing stimuli might also have other impacts, where compensatory actions can be beneficial. Taking into account a pneumatic bending actuator, following a healing process, the actuator might regain its full bending angle (correlated with the recovery of material fracture points), albeit requiring a lower pressure input to achieve it (associated with the restoration of the Young's modulus).^[^ ^37^ ^]^ Depending on the kinetics of the self‐healing materials (reaction rate), achieving full recovery of the mechanical properties can take time, and in some cases, it can be on the order of a few days.^[^ ^12^ ^]^ This means that the application of healing stimuli, which typically softens the material by breaking more chemical bonds, can compromise the robot's function, such as lowering its load exertion ability.^[^ ^30^ ^]^ To address this issue and recover the robot's function, Kashef et al. have employed active force compensation using a layer‐jamming‐based variable stiffness mechanism.^[^ ^30^ ^]^ In this scenario, the self‐healing chamber within the layer‐jamming system can restore its airtightness, enabling the active compensation of the load‐bearing capacity through variable stiffness. Load‐bearing capacity can be enhanced by other various variable stiffness mechanisms,^[^ ^223^, ^224^ ^]^ all of which have the potential to counteract the softening effect of stimuli in self‐healing soft robots.

### 6.2 Technologies for Healing Assessment

To assess self‐healing at the material level, destructive methods are often used to evaluate the restoration of initial material properties post‐healing.^[^ ^13^ ^]^ This involves mechanical recovery, commonly analyzed through tensile or bending testing conducted before damage and after self‐healing. These tests generate fracture stresses and strains, which enable calculation of the healing efficiency.^[^ ^12^, ^13^ ^]^ Alternatively, nondestructive methods, such as widespread use of microscopic images^[^ ^136^ ^]^ or ultrasonic pulse analysis,^[^ ^37^ ^]^ are also employed. Acoustic emission is another nondestructive approach investigated by Bekas et al.^[^ ^225^ ^]^ However, while such nondestructive methods offer insight into the physical condition of damaged surfaces (indicating the sealing of the damage), they do not provide information about the reformation of (physico)chemical bonds on the molecular level. Bond reformation would be monitored using other techniques such as Fourier transform infrared spectroscopy.^[^ ^136^ ^]^

Most of the methods discussed for evaluating mechanical performance restoration after healing at the specimen level are impractical for assessing soft robots due to their intricate geometry. As we progress toward autonomous healing in (soft) robotics, self‐assessment becomes advantageous.^[^ ^38^ ^]^ Many of the damage detection mechanisms discussed in Section 2.2, which rely on self‐healing sensors, can be utilized for healing assessment. This parallels the human body, where the nervous system often detects damage but also monitors healing. An example can be real‐time tracking of the resistance of integrated self‐healing sensors or heaters.^[^ ^29^, ^226^ ^]^ While damage can be detected as a sharp change in resistance, the healing progress can be estimated based on the recovery of the initial resistance. Such a real‐time monitoring facilitates prompt action if recovery deviates from expectations. Nevertheless, after the healing, it remains a challenge to distinguish whether the change in baseline resistance is due to poor healing/misalignment or other factors, like environmental effects or healing stimuli.^[^ ^76^ ^]^ This can potentially require human intervention, which is undesirable. One autonomous approach is to employ multiple healing assessment systems. Considering a sensorized bending actuator, the recovery of the sensor, as well as the bending performance of the actuator monitored with a camera, can be utilized to better assess the system's health.^[^ ^38^ ^]^ Furthermore, Terryn et al. demonstrated how healing in an electronic skin can be evaluated by employing a robot manipulator to probe on top of the conductive traces of the skin, where poor healing results in disruption of the conductive pathway.^[^ ^76^ ^]^

Another approach to monitor healing is by using tomography analysis by electrical impedance measurements^[^ ^67^, ^80^ ^]^ (Figure 2f) or inferred tomography (using an Infrared camera) to monitor temperature distribution in a self‐healing (conductive) material^[^ ^29^ ^]^ (Figure 2c),^[^ ^225^ ^]^ similar to medical imaging for human health monitoring. Effective healing will show no more concentration of electrical impedance or heat distribution. Moreover, Karshalev et al. evaluated the healing of their robotic swimmer, assessing how well the two separated pieces become aligned by the magnetic field, by studying the magnetic hysteresis.^[^ ^123^ ^]^ In samples that are well‐aligned, magnetic properties exhibit a pronounced directional dependence, similar to the characteristics observed in anisotropic magnetic materials.^[^ ^227^, ^228^ ^]^

While self‐healing soft robots still depend heavily on human intervention and external devices to assess their recovered functionality, their autonomy could be significantly enhanced by integrating advanced multimodal sensory mechanisms, vision‐based data acquisition, and artificial intelligence for sophisticated data processing.

## 7 Combinations, Challenges, and Future Advances

In real‐world scenarios, the location, type, and severity of damage are often uncertain. Furthermore, external assistance and required facilities may not always be readily available, leading to increased costs in terms of energy, money, and time. For instance, consider a soft actuator used as a finger in a prosthetic hand. Daily activities take place in highly dynamic environments, exposing the actuator to varying types of damage. Maintaining the damage in a closed state during the treatment process can be challenging. Additionally, disassembly and reassembly of the finger can be burdensome for the user. Providing stimuli, such as heating through an oven, necessitates an external device, and ensuring the effectiveness of the healing process often relies on manual or visual inspection, which can be unreliable.

**Figure 7:**
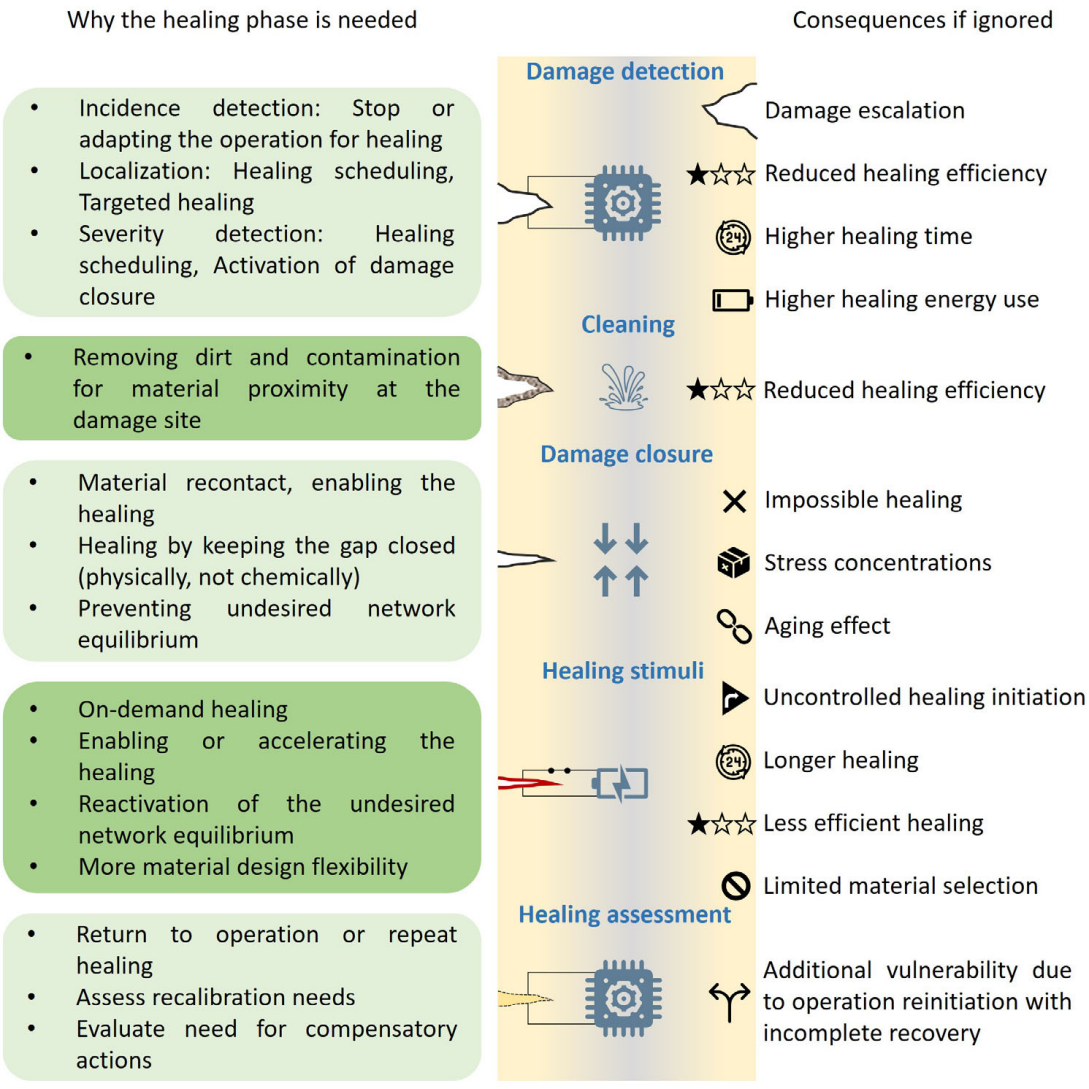
This summary highlights the necessity of each healing phase and explores the potential consequences of omitting any phase. These insights help in selecting the appropriate phases and technologies tailored to specific applications. Notably, the discussion of the fourth phase emphasizes the advantages of integrating a healing stimulus. However, the authors remain neutral regarding the choice between autonomous self‐healing materials (requiring no external triggering) and nonautonomous ones. The decision depends entirely on the application, the type of damage most likely to occur, and the environmental conditions.

Integrating all healing phases into the design can significantly enhance the robustness of robots, enabling them to recover from diverse damage conditions with minimal or no external assistance. However, the inclusion of technologies for each healing phase must be tailored to the specific application. The application determines the selection of a self‐healing material, the potential types and characteristics of damage, and the environmental conditions, all of which are critical for deciding which healing phases to incorporate. The importance of each phase and its specific relevance to different scenarios and the consequences of omitting a phase have been detailed in the previous five sections. This information is summarized in **Figure** **7**, providing a comprehensive guide for designing self‐healing systems.

**Figure 8:**
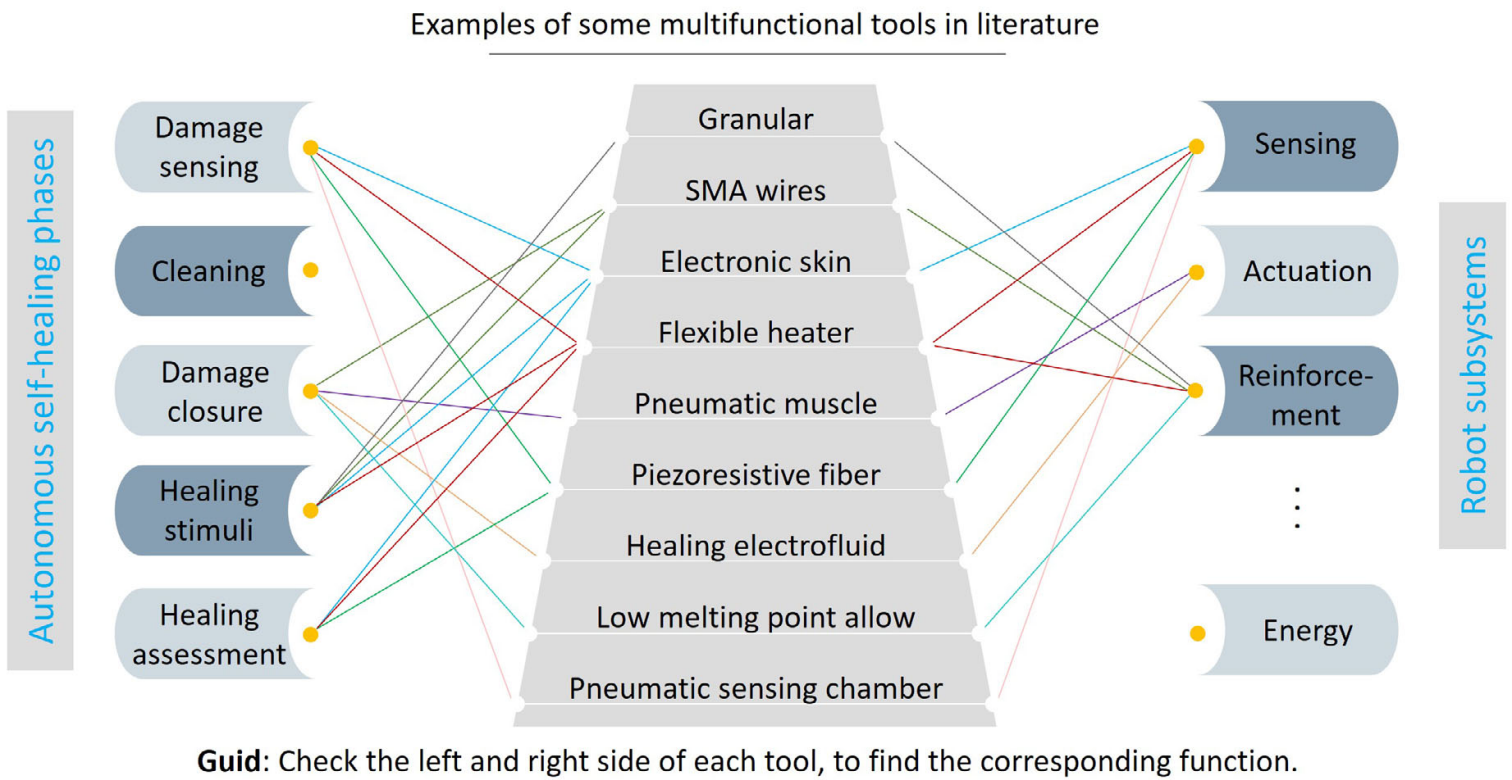
Examples of some tools with multifunctional purposes (middle), including at least one autonomous healing phase (left) and one subsystem in a robot (right). Granular,^[^ ^49^ ^]^ SMA wire,^[^ ^37^ ^]^ electronic skin,^[^ ^48^ ^]^ flexible heater,^[^ ^29^ ^]^ pneumatic muscle,^[^ ^53^ ^]^ piezoresistive fibers,^[^ ^38^ ^]^ healing electrofluids,^[^ ^48^ ^]^ low‐melting‐point alloys,^[^ ^168^ ^]^ pneumatic sensing chamber.^[^ ^87^ ^]^

There are two primary concerns regarding the inclusion of technology for each autonomous healing phase: the potential escalation of system complexity and increased energy consumption. While there are several applications and scenarios where not all phases require an additional system to be added to the robot, introducing even one more hardware system would significantly complicate the design and processing of the robots. One approach to tackle this issue is to implement hardware multifunctionality.^[^ ^229^ ^]^ Throughout the manuscript, several cases of possible multifunctionality have been mentioned. For example, the combination of stimuli‐responsive self‐healing materials and stimuli‐responsive actuators offers dual functionality.^[^ ^22^, ^24^ ^]^ Additionally, multi‐stimuli responsive materials used for actuation,^[^ ^230^ ^]^ and materials that can be healed under different environments,^[^ ^157^ ^]^ show promise to facilitate multifunctionality. However, it should be noted that stimuli‐responsive actuators currently exist in small‐scale structures, operate slowly, and have been tested in environments with human‐controlled stimulation.^[^ ^22^, ^24^ ^]^ These limitations suggest areas for future research and improvement. The work of Kashef Tabrizian et al. demonstrates another multifunctionality where SMA wires are used as reinforcement, damage closure agent, and healing triggering.^[^ ^37^ ^]^ Damage sensors can potentially be used for health assessment of the system. A heat source, potentially integrated, could function as actuation,^[^ ^112^ ^]^ triggering damage closure,^[^ ^37^ ^]^ healing,^[^ ^111^ ^]^ and also autonomic perspiration for self‐cleaning.^[^ ^110^ ^]^ **Figure** **8** provides an overview of some tools with multifunctional purposes utilized in the literature. In the authors’ view, any technology intended for an autonomous healing phase is more compelling when contributing to the robot's main operation (Figure 8). This approach allows multifunctionality to be explored not only across different healing phases but also between the healing phases and the robot's primary subsystems (Figure 8).

Furthermore, adding more components to a robotics system would result in increased energy consumption, either for the activation of added assistive healing mechanisms or their passive integration, leading to increased weight and size of the robot, ultimately requiring more energy. Although this is of less concern for tethered applications, it can be a serious issue for untethered applications. Aubin et al. have delved into enduring robots from an energy standpoint,^[^ ^231^ ^]^ introducing various embodied energy strategies such as energy source multifunctionality,^[^ ^231^ ^]^ e.g., batteries can serve as the body of the robot,^[^ ^232^ ^]^ or combining power and actuation.^[^ ^233^ ^]^ In future, it is expected to see more self‐healing soft and multifunctional energy harvesters and energy storage devices integrated in robots.^[^ ^234^ ^]^ Using autonomous self‐healing materials that do not require triggering for healing or utilizing natural sources for healing triggering can reduce energy consumption as well. Water, sun, and air can serve as examples of sources to trigger healing. Robots can be constructed from local materials, such as icy robots designed for icy planets.^[^ ^235^ ^]^ There are other principles found in nature that can be further refined and applied in the development of self‐healing and damage‐resilient soft robots. An example is real‐time material synthesis with the assistance of natural sources, such as in hydrogels where water is a primary substance.

Most of the currently proposed solutions for enabling autonomous healing in robotics, such as damage sensors, closure agents, or integrated stimuli providers, are discreetly integrated and effective for specific locations of the robots under certain damage conditions. These technologies demonstrate to be reliable and perform well in applications where the environment is fixed, and tasks are predesigned for the robot. In such cases, healing‐assistive mechanisms can be designed and implemented based on well‐known damage risks. However, as we transition toward untethered soft robots with a diversity of tasks similar to those performed by humans, the necessity for full‐body nociception and the complete embodiment of other healing‐assistive mechanisms becomes significant. Electronic skins, such as EIT sensors and pneumatic sensing chambers, offer promising solutions toward achieving full‐body awareness. In embodying other healing‐assistive mechanisms, inspiration can be drawn from the human body, which is a mix of fluids and solids. This would enhance the reliability of gap filling and healing. This is why the authors believe that new combinations of intrinsic and extrinsic self‐healing materials should be taken into account. This combination does not necessarily depend on creating new materials but can be explored at the system level. Using liquid–solid or solid–liquid phase transition can assist in this regard.^[^ ^32^, ^48^, ^119^, ^168^, ^236^ ^]^

## 8 Conclusion

The rapidly advancing research in soft robotics will lead to a future where this new generation of robots will play a much more significant role in both industry and daily life. From the authors’ viewpoint, high‐potential beachhead markets for soft robots include agricultural robots, prosthetics and orthotics, and the fast‐growing field of humanoids. However, the broader adoption of soft robots underscores the need for a heightened focus on enhancing their resilience and longevity.

The introduction of healing features to soft robots can extend their lifespan, thereby increasing their economic competitiveness and sustainability. However, there remains significant progress to be made in developing reliable healing technologies tailored to specific applications and environmental conditions. Such advancements are essential to convince a broader audience to adopt self‐healing technologies instead of the current non‐sustainable polymers. One of the primary steps in overcoming the challenges in healing technologies is to focus on standardized processing techniques for self‐healing materials, as well as establishing clear standards for characterizing the healing.^[^ ^237^ ^]^ Collaborating with industries can help researchers better understand real‐world needs and guide the development of self‐healing systems toward making them commercially viable.^[^ ^30^ ^]^ Another challenge with many current self‐healing materials for robotics is their lack of stable mechanical properties and susceptibility to creep. Researchers are addressing this issue by incorporating dual‐network structures.^[^ ^238^ ^]^ There are other challenges with the current self‐healing materials such as lack of growability and extended healing times.

Nevertheless, several challenges associated with self‐healing materials can be mitigated through external assistance. Currently, the process of self‐healing in soft robotics often relies heavily on human intervention and the use of external devices to boost the healing. This significant dependence on human involvement poses a major challenge, particularly in applications requiring autonomous operation, such as robotic systems functioning in remote or inaccessible environments. For such systems to reach their full potential, it is crucial to minimize or eliminate external involvement in the healing process. In this work, we aimed to address the critical issue of human dependency in the healing process by exploring healing‐assistive technologies at five consecutive phases, thereby advancing toward full autonomy in self‐healing soft robotics. It is important to consider that the proposed assistive technologies should either be self‐healing and resilient to damage or at least designed and integrated in a way that is less susceptible to damage. In real‐world scenarios, damage can occur multiple times and possibly at the same location.

Last, it should be noted that damage resilience in soft robotics is not limited to use of self‐healing materials. Robots can benefit from damage adaptive strategies.^[^ ^239^, ^240^ ^]^ For example, a multi‐pedal robot could adjust its walking pattern in response to damage that causes a loss of function in one of its limbs,^[^ ^239^ ^]^ via software adaptation. The recent work of Yang et al. shows hardware‐level adaptation, a soft quadruped robot that can self‐amputate its body when an issue arises, thanks to the use of a reversible cohesive interface between its body parts.^[^ ^241^ ^]^ Damage‐responsive valves have been utilized to enable robots to maintain functionality when damage occurs.^[^ ^57^, ^242^ ^]^ Furthermore, modular and reconfigurable soft robotic systems enable the repair or replacement of modules upon damage.^[^ ^243^, ^244^, ^245^ ^]^ Nevertheless, autonomous reconfiguration remains a challenge and is highly dependent on damage detection and localization. In this context, the work of Wang et al. on combining liquid metals with magneto‐activation shows promise for autonomous shape‐shifting.^[^ ^236^ ^]^ One promising research direction to improve the damage resilience of soft robots involves combining autonomous self‐healing capabilities with damage adaptation strategies.

## Conflict of Interest

The authors declare no conflict of interest.

## Author Contributions

**Seyedreza Kashef Tabrizian**: conceptualization: (equal); investigation: (lead); methodology: (lead); project administration: (lead); visualization: (lead); writing—original draft: (lead); writing—review & editing: (lead). **Seppe Terryn**: conceptualization: (equal); funding acquisition: (supporting); investigation: (supporting); methodology: (supporting); project administration: (supporting); supervision: (equal); writing—review & editing: (equal). **Bram Vanderborght**: conceptualization: (supporting); funding acquisition: (lead); investigation: (supporting); methodology: (supporting); project administration: (supporting); supervision: (lead); writing—review & editing: (equal).
